# Genome-wide investigation of WRKY gene family in *Lavandula angustifolia* and potential role of *LaWRKY57* and *LaWRKY75* in the regulation of terpenoid biosynthesis

**DOI:** 10.3389/fpls.2024.1449299

**Published:** 2024-10-08

**Authors:** Kelaremu Kelimujiang, Wenying Zhang, Xiaxia Zhang, Aliya Waili, Xinyue Tang, Yongkun Chen, Lingna Chen

**Affiliations:** ^1^ Xinjiang Key Laboratory of Special Species Conservation and Regulatory Biology, Key Laboratory of Plant Stress Biology in Arid Land, School of Life Sciences, Xinjiang Normal University, Urumqi, China; ^2^ Key Laboratory of Plant Resources, Institute of Botany, Chinese Academy of Sciences, Beijing, China

**Keywords:** *Lavandula angustifolia*, WRKY gene family, expression analysis, functional identification, terpenoid biosynthesis

## Abstract

The WRKY transcription factors are integral to plant biology, serving essential functions in growth, development, stress responses, and the control of secondary metabolism. Through the use of bioinformatics techniques, this research has effectively characterized 207 members of the WRKY family (LaWRKY) present in the entire genome of *Lavandula angustifolia*. Phylogenetic analysis classified LaWRKYs into three distinct categories based on conserved domains. Collinearity analysis revealed tandem repeats, segmental duplications, and whole genome duplications in *LaWRKYs*, especially for segmental duplication playing a significant role in gene family expansion. *LaWRKYs* displayed distinct tissue-specific expression profiles across six different tissues of *L. angustifolia*. Particularly noteworthy were 12 genes exhibiting high expression in flower buds and calyx, the primary sites of volatile terpenoid production, indicating their potential role in terpenoid biosynthesis in *L. angustifolia*. RT-qPCR analysis revealed a notable increase in the expression levels of most examined *LaWRKY* genes in flower buds in response to both intense light and low-temperature conditions, whereas the majority of these genes in leaves were primarily induced by drought stress. However, all genes exhibited downregulation following GA treatment in both flower buds and leaves. Overexpression of *LaWRKY57* (La13G01665) and *LaWRKY75* (La16G00030) in tobacco led to a reduction in the density of glandular trichomes on leaf surfaces, resulting in changes to the volatile terpenoid composition in the leaves. Specifically, the content of Neophytadiene was significantly elevated compared to wild-type tobacco, while compounds such as eucalyptol, cis-3-Hexenyl iso-butyrate, and D-Limonene were produced, which were absent in the wild type. These findings provide a valuable reference for future investigations into the biological functions of the *WRKY* gene family in *L. angustifolia*.

## 1 Introduction

Transcription factors, a group of proteins governing gene expression, play a crucial role in the evolution and adaptation of complex functions, including metabolism, cell morphogenesis, signal transduction, and responses to environmental stress (Song and Gao, 2014). Among these, the WRKY family stands out as one of the most extensively researched and representative groups of transcription factors in higher plants (Yu et al., 2024). The DNA-binding structural domain of WRKY is characterized by the highly conserved WRKYGQK motif and one or two conserved structural domains of approximately 60 amino acid residues (Song and Gao, 2014). Specifically, the N-terminus of WRKY is associated with DNA binding, containing the conserved WRKYGQK sequence, while the C-terminus is involved in protein interactions and auxiliary DNA binding, typically comprising CX_4_-7CX_22_-23HXH/C (Wei et al., 2016). WRKY transcription factors bind to their homologous (C/T)TGAC(C/T) W-box cis-elements to regulate target gene expression, performing crucial regulatory functions (Yu et al., 2024). These transcription factors are categorized into three major groups based on the number and variations of their structural domains. Group I possesses two conserved WRKY domains and a C_2_H_2_ (CX_4_-5CX_22_-23HXH) type zinc finger structure; Group II contains one WRKY domain and a C_2_H_2_ type zinc finger structure, further divided into five subcategories (IIa, IIb, IIc, IId, and IIe) based on amino acid sequence differences; and Group III comprises one WRKY domain and a C_2_HC (CX_7_CX_23_HXC) type zinc finger structure, exclusively identified in higher plants (Chen et al., 2020).

In recent years, WRKY gene families have been documented in various plant species, such as *Arabidopsis thaliana* (70) (Wang et al., 2011), *Populus trichocarpa* (71) (Song and Gao, 2014), *Camellia sinensis* (56) (Wang et al., 2019), *Eucommia ulmoides* (45) (Liu et al., 2021), and *Cannabis sativa* (48) (Yu et al., 2024). As more WRKY genes are identified, their functions have become clearer, encompassing leaf senescence (Han et al., 2014), glandular trichomes development (Pesch et al., 2014), biosynthetic secondary metabolites (Chen et al., 2017; Yu et al., 2024), seed dormancy (Zhou et al., 2020) and germination (Wang et al., 2021), etc. Furthermore, there is a growing evidence suggesting that specific WRKY proteins play a constructive role in the regulation of secondary metabolite synthesis and accumulation in plants. A notable illustration is WRKY1, which regulates terpenoid synthesis by modulating the expression levels of *ORCA2/3*, *CrMYC2*, *ZCTs* in *Catharanthus roseus*, and *CAD1-A* in *Gossypium arboreum*, thereby influencing the synthesis of monoterpenes or sesquiterpenes (Xu et al., 2004; Suttipanta et al., 2011). In *Artemisia annua*, the induction of WRKY1 by MeJA, combined with the W-box cis-acting element of the ADS promoter, results in the activation of *CYP71AV1* transcription, thereby promoting the synthesis of artemisinin (Jiang et al., 2016). Additionally, the overexpression of *TcWRKY8* and *TcWRKY47* significantly enhances the expression level of genes associated with paclitaxel biosynthesis in *Taxus chinensis* (Zhang et al., 2018). Furthermore, *Zea mays ZmWRKY79* positively regulates the expression of terpenoid-synthesis genes, contributing to the maize response to pathogen stress (Fu et al., 2017). In summary, WRKY transcription factors not only play a positive role in regulating the biosynthesis and accumulation of secondary metabolites in plants but also interact with various signaling pathways, participating in plant growth, development, and stress responses.

*Lavandula angustifolia*, a member of the Lamiaceae family, comprises a diverse group of 39 species, and its essential oils are extensively utilized (Guo et al., 2020). The EOs derived from *L. angustifolia* possess substantial economic importance and are widely applied in industries such as perfume, cosmetics, antibacterial products, antioxidants, anti-anxiety formulations, and other medical applications (Sarker et al., 2013). These oils are primarily composed of monoterpenes, including linalool, camphor, and 1,8-cineole, with a minor presence of sesquiterpenes (Lane et al., 2010). Glandular trichomes, epidermal secretory structures of the plant, serve as the storage site for these compounds (Li et al., 2021). However, the lack of information on relevant genes, particularly plant-specific transcription factors such as WRKY transcription factors, has led to a gap in understanding their roles in the growth, development, or response to biological stress in *L. angustifolia*. This study successfully identified a total of 207 *LaWRKY* genes from the *L. angustifolia* genome. These genes were classified into three distinct groups, and their phylogenetic relationships, chromosome localization, intra-species repeatability analysis, and expression profiles under different stresses were comprehensively examined and analyzed. Furthermore, the preliminary functions of two prominent genes, *La13G01665* and *La16G00030*, have been identified. The findings of this study carry significant value for future research on the *WRKY* gene in *L. angustifolia*.

## 2 Materials and methods

### 2.1 Identification of LaWRKY family members

WRKY protein sequences from Arabidopsis (*Arabidopsis thaliana*), rice (*Oryza sativa*), poplar (*Populus euphratica*), and tomato (*Solanum lycopersicum*) were obtained from the Phytozome V12.1 website (http://www.phytozome.net/). The HMM pattern (PF03106) of the WRKY domain was acquired from the Pfam database. Utilizing *L. angustifolia* genome data (Li et al., 2021) and local BLAST alignment, the LaWRKY protein sequences were identified. To confirm the presence of the WRKY domain in the obtained sequences, the domain structure of LaWRKY proteins was validated using the NCBI domain database search program (https://www.ncbi.nlm.nih.gov/Structure/cdd/wrpsb.cgi) in conjunction with the HMMER HMM Search program. Genes lacking the WRKY domain were excluded. The ExPASY PROT-PARAM tool (http://web.expasy.org/protparam/) was employed to calculate the physical and chemical properties of each gene, including molecular weight (MW) and isoelectric point (pI). Subcellular localization was predicted using the WoLF PSORT website (https://wolfpsort.hgc.jp/).

### 2.2 LaWRKY sequence alignment and phylogenetic tree construction

The LaWRKY protein sequences were aligned using DNAMAN 7.0 software, and the conservative structural domains were analyzed using the online software MEME (multiple Em for Motif Elicitation) (http://meme.nbcr.net/meme/intro.html) with the following parameters: Maximum width: 50, Minimum sites: 2, p < 0.05. The resulting analysis was visualized using TBtools (v1.098765). Additionally, LaWRKY amino acid sequences underwent multiple sequence alignments using MEGA10.0 software. A phylogenetic tree, consisting of 1000 bootstrap repeats, was generated using the neighbor-joining (NJ) method, with *Arabidopsis* as a model plant (Baillo et al., 2020) and LaWRKY as references. The phylogenetic tree was further enhanced using the online software iTOL (http://itol.embl.de/).

### 2.3 LaWRKY chromosome localization and intra-species collinearity analysis

The precise genomic positions of *LaWRKY* genes in the *L. angustifolia* genome were determined using chromosome positioning in TBtools (v1.098765). Homology analysis was conducted on the protein sequences of *LaWRKY*, and genes with a fragment identity greater than 79.99% were selected for collinearity analysis to investigate gene duplication events.

### 2.4 LaWRKY expression profile and promoter cis-acting elements prediction

**Figure 1:**
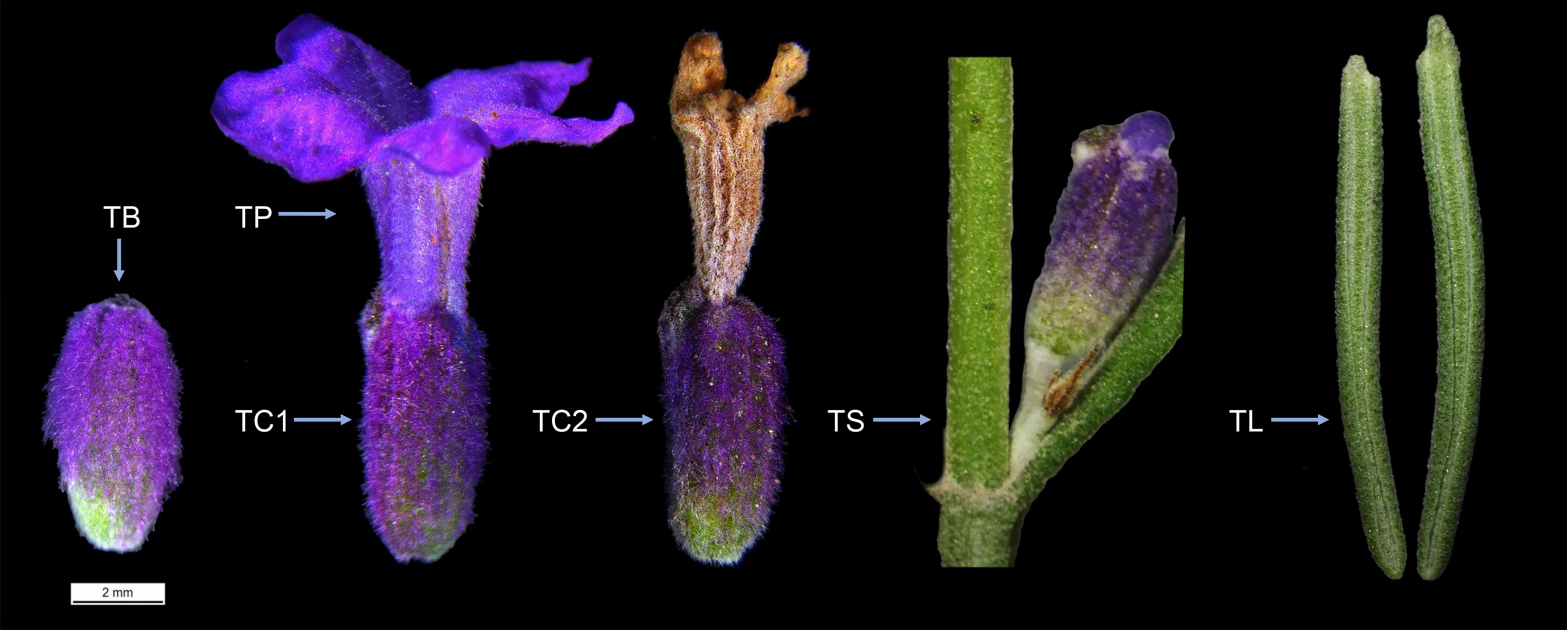
Schematic diagram of different tissues of *L. angustifolia*. TB, bud; TP, petal; TC1, fresh calyx; TC2, mature calyx; TS, stem; TL, leaf.

The *L. angustifolia* cultivar utilized in this study was ‘Jieyou 6’, which was obtained from the campus of Xinjiang Normal University. RNA-seq was employed to examine gene expression profiles in different tissues of ‘Jieyou 6’, including bud (TB), petal (TP), fresh calyx (TC1), mature calyx (TC2), stem (TS), and leaf (TL) (**Figure 1**). RNA-seq data were deposited in GenBank with index number PRJNA892961. The sequence of the 2000 bp region upstream of the start codon of *LaWRKY* genes was extracted from *L. angustifolia* genome sequencing data (Li et al., 2021). The PlantCare website (http://bioinformatics.psb.ugent.be/wetools/plantcare/html/) was used to predict cis-acting elements in gene promoter regions. Finally, the TBtools software was utilized to generate a cis-regulatory element diagram of the promoter.

### 2.5 RT-qPCR analysis of gene expression under different hormone and stress conditions

The *L. angustifolia* cultivar ‘Jieyou 6’ at Xinjiang Normal University was cultivated under standardized conditions, with a plant spacing of 60 cm and a row spacing of 80 cm. Since 2021, these plants have consistently exhibited regular blooming each summer. Uniformly growing branches were selected and treated with 100 μM gibberellin (GA), 100 μM abscisic acid (ABA), 100 μM methyl jasmonate (MeJA), and 100 μM ethylene (Eth). Samples were collected 24 hours post-treatment (Sun et al., 2018). As a control for hormone treatments, buds and leaves were sprayed with sterile water and subsequently harvested. Two distinct light conditions, specifically 40,000 lux and complete darkness within an incubator, were implemented. Low-temperature treatment was conducted at 4°C, while drought stress was induced by irrigating seedlings without sterile water. Bud and leaf samples were harvested 12 hours after treatment initiation, rapidly frozen in liquid nitrogen, and subsequently stored at -80°C.

RNA was extracted from flower buds and leaves of *L. angustifolia* using the RNAprep Pure Plant Kit (Tiangen Biochemical Technology Company, Beijing). Subsequently, the extracted RNA was reverse transcribed into cDNA using the PrimeScript™ RT reagent Kit with gDNA Eraser from Takara Biomedical Technology (Beijing). *LaActin* served as the internal reference, and the RT-qPCR assay for 10 *LaWRKY* genes was performed using TB Green Premix Ex Taq II (Takara Biomedical Technology, Beijing). Three biological replicates were established for each sample, with cDNA serving as the template. The primer sequences utilized in the assay are provided in **Supplementary Table 1**. The reaction system comprised a 20 µL volume, including 1 µL of diluted cDNA template, 0.4 µL of each upstream and downstream primer, 10 µL of Power SYBR ^®^ Green PCR Master Mix (2×), and 8.2 µL of ddH2O. The PCR reaction program involved an initial pre-denaturation step at 95°C for 30 seconds, followed by 40 cycles of denaturation at 95°C for 5 seconds and annealing at 60°C for 20 seconds. The fluorescence value change curve and melting curve were analyzed, and the expression level was calculated by 2^-ΔΔCT^ method (Livak and Schmittgen, 2001).

### 2.6 Cloning and functional analysis of *LaWRKY57* (La13G01665) and *LaWRKY75* (La16G00030) genes

After conducting a comparative analysis on the NCBI website, it was determined that the genes *La13G01665* and *La16G00030* exhibit homologies of 52.5% and 69.95% with *Salvia miltiorrhiza WRKY57* and *WRKY75*, respectively, within the Lamiaceae family. Consequently, these genes have been designated as *LaWRKY57* and *LaWRKY75*.

Using a combination of cDNA samples extracted from various lavender tissues as a template, gene fragments of *LaWRKY57* and *LaWRKY75* were amplified utilizing primers EYFP1665 and EYFP0030 (**Supplementary Table 2**), respectively, in a reaction system totaling 25 μL. This system included 12.5 μL of 2x Taq PCR Mix, 1.0 μL each of upstream and downstream primers, 1.0 μL of cDNA template, and 10.5 μL of ddH_2_O. The PCR program consisted of an initial denaturation step at 95°C for 5 minutes, followed by 35 cycles of denaturation at 95°C for 15 seconds, annealing at 58°C for 15 seconds, extension at 72°C for 90 seconds, and a final extension step at 72°C for 5 minutes. The amplified products were ligated into the pSAT6-nEYFP-N1 vector using a seamless cloning kit from Shanghai Shenggong, Shanghai, China, following the manufacturer’s instructions. Subsequently, the plasmid was subjected to detection with EYFPdetc primers and sequencing (**Supplementary Table 2**) before being extracted and prepared for subcellular localization observation in lettuce (Zhang et al., 2021).

Utilizing identical cDNA templates, gene fragments of *LaWRKY57* and *LaWRKY75* were amplified with primers LC1665 and LC0030, respectively (**Supplementary Table 2**). Employing consistent system parameters, reaction programs, and vector construction techniques, the two genes were integrated into the PEZR(K)-LC vector and subsequently introduced into *E. coli* DH5α. Following confirmation through LCdete primers and sequencing validation (**Supplementary Table 2**), the recombinant plasmid was transferred into Agrobacterium EHA105 to generate a recombinant strain. Tobacco underwent heterologous transformation through the Agrobacterium-mediated leaf disc method and was subsequently screened with Kanamycin. The transgenic positive plants were distinguished through RT-qPCR analysis (Chen et al., 2023). Glandular trichomes on the leaves of the transgenic strains were examined using scanning electron microscopy, and the volatile products of leaves were analyzed via gas chromatography/mass spectrometry (GC/MS), providing an initial assessment of the gene’s function.

## 3 Results

### 3.1 Physicochemical properties and subcellular localization of LaWRKY protein

In this study, a total of 207 LaWRKY proteins were identified, and their physicochemical properties and phylogenetic grouping are presented in **Supplementary Table 3**. The size of the LaWRKY proteins ranged from 97 amino acids (La05G01130) to 1038 amino acids (La22G01781), displaying a wide and uneven length distribution. The average size of these proteins was 328.11 amino acids, indicating structural and functional variation. The molecular weight ranged from 11.1 kDa (La05G01130) to 114.4 kDa (La22G01781), while the isoelectric point (pI) ranged from 4.70 (La18G01763) to 10.71 (La05G01126). Among these proteins, 106 LaWRKY proteins were classified as acidic (pI < 7.0). Subcellular localization analysis indicated that the predominant localization of LaWRKY proteins was within the nucleus, with specific distribution in subgroups I, IIa, IIe, and all the members of subgroup III. Furthermore, four members of subgroup IIc and La20G01890 from subgroup IIb were localized in chloroplasts, while three members of subgroup IIc and La12G00032 from subgroup IId were identified in the cytoplasm. Additionally, La05G01130 from subgroup IId was localized in the mitochondria, and La04G00827 from subgroup IIb was situated in the vacuole.

### 3.2 Classification and phylogenetic analysis of *LaWRKY* genes

The sequence alignment analysis identified two distinct structural motifs within the WRKY protein (**Supplementary Figures 1, 2**), which formed the foundation for its classification. Firstly, with respect to the WRKYGQK domain, 193 out of 207 LaWRKY proteins exhibited highly conserved WRKYGQK sequences, while the remaining 13 members displayed four mutations (WRKYGKK, WHKCGMN, WRKYGPK, and WRKYGQI). The 35 members possessing two WRKY domains were classified as Group I, whereas LaWRKY members with a single WRKY domain were assigned to Groups II and III. Secondly, the classification of Groups II and III was based on the presence of the zinc finger motif. Specifically, 142 LaWRKYs with the C2H2 type zinc finger motif were assigned to Group II, while the remaining 18 with the C2HC zinc-finger motif were categorized under Group III. Notably, except for 9 LaWRKYs in group II lacking one H or C, the remaining members exhibited a complete structural composition.

**Figure 2:**
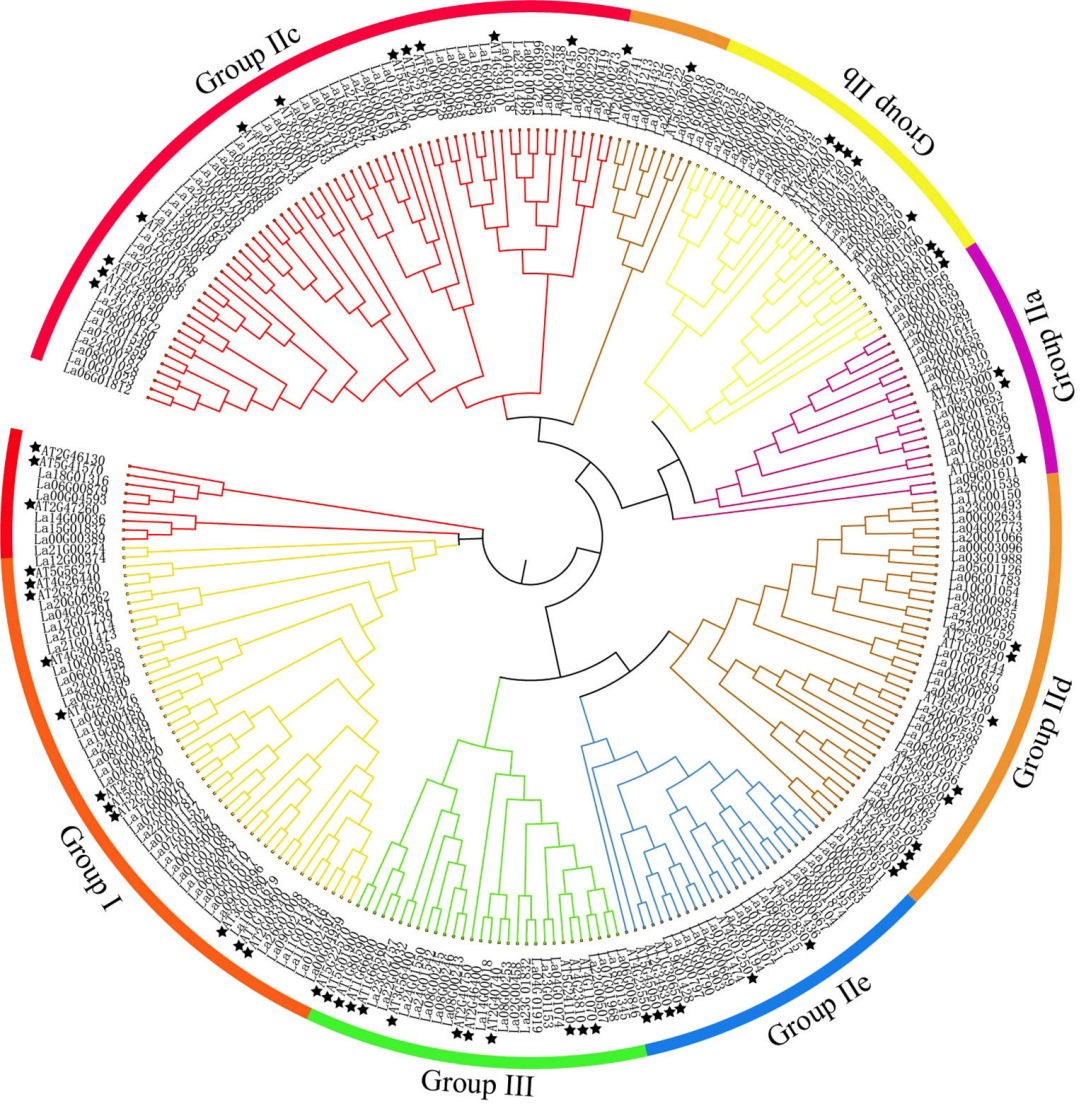
Phylogenetic tree of WRKY domains in *L. angustifolia* and *Arabidopsis.* Stars symbolize Arabidopsis WRKY members, with varying colors denoting distinct classifications within the WRKY family.

A phylogenetic tree was constructed using 207 LaWRKY and 64 AtWRKY (Baillo et al., 2020) (**Figure 2**). The WRKY proteins were categorized into three groups (group I, II, and III), confirming the findings from the sequence alignment. Group II was further divided into five subgroups (a, b, c, d, and e), aligning with the classification of AtWRKY. Particularly, group IIc exhibited the highest number of LaWRKY members (58), while AtWRKY had only 10 members in this subgroup. The subsequent subgroups in descending order were IId with 38 members, IIb with 20 members, IIe with 19 members, and IIa with 16 members. From an evolutionary standpoint, it is evident that Group IIa and IIb, as well as IId and IIe, demonstrate a high degree of relatedness, indicating a shared ancestral origin for these two WRKY groups. Additionally, Groups III, IId, and IIe formed distinct branches, indicating instances of gene replication and divergence among these members. Notably, a total of five pairs of LaWRKY and AtWRKY were clustered together in groups I, IIc, IId, and III, while the remaining LaWRKY members initially clustered separately before eventually gathering with AtWRKY. This finding suggests significant conservation among family members of the same species. Based on the genetic relationships, it can be inferred that AtWRKY exhibits an earlier onset of divergence compared to LaWRKY, indicating that *L. angustifolia* underwent evolutionary development subsequent to *Arabidopsis*.

### 3.3 Gene structure and conserved domain analysis of the LaWRKY family

**Figure 3:**
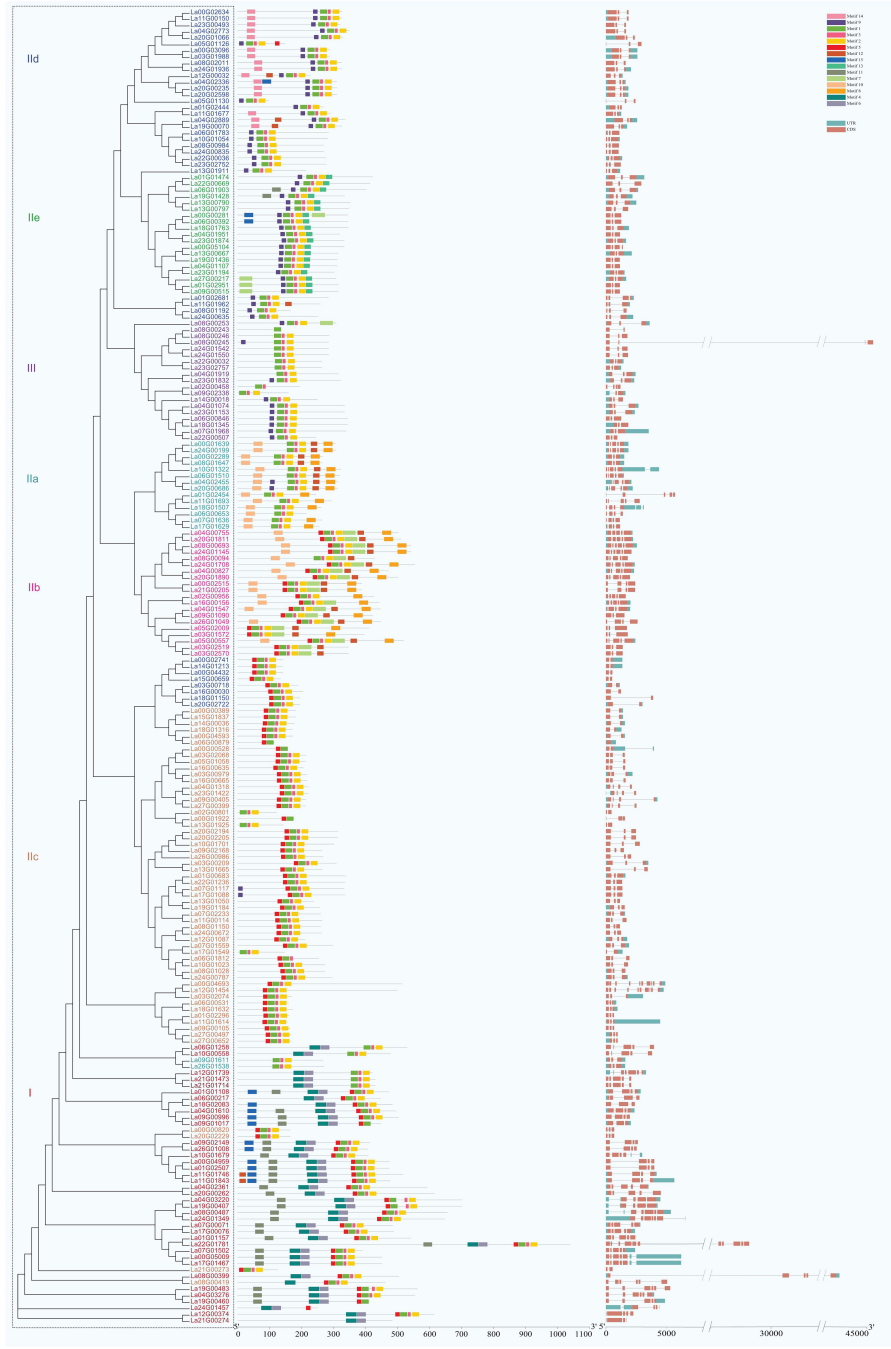
The intron-exon structure of LaWRKY genes. Exons and introns are visually represented by orange-yellow rectangles and single lines, respectively. The conserved motifs of LaWRKY proteins are denoted by boxes of varying colors, each representing a distinct motif and its respective position within the WRKY sequence. The motif tag for LaWRKY proteins is also indicated.

The structural analysis of *LaWRKY* genes revealed that closely related members within the same group exhibited similar exon-intron structures and numbers. The number of introns in *LaWRKY* genes varied from 0 to 10, with 111 (53.6%) *LaWRKY* genes containing two introns (**Figure 3**). Additionally, 33 (15.9%) and 32 (15.4%) genes possessed 4 and 3 introns, respectively, while 19 (9.1%) had 1 intron. The remaining 10 (4.8%) genes had 0, 5, 6, 9, and 10 introns. Notably, groups IIe and III predominantly consisted of genes with 2 introns, while groups IIa and IIb primarily contained genes with four introns. Group IId genes, on the other hand, were mostly characterized by the presence of a single intron.

The MEME online software analysis identified 15 conserved motifs within the LaWRKY family (**Figure 3**). Each LaWRKY member harbored between 1 and 9 motifs, with motif 1 and 2 being universally present in all LaWRKY members. Furthermore, distinct motifs characterized each group. Specifically, motifs 4, 6, and 11 exclusively occurred in group I, motif 7 solely appeared in group IIb, motif 14 was exclusive to group IId, and motif 13 was only observed in group IIe. These conserved motifs, unique to certain groups, may play a role in specific physiological responses.

### 3.4 Chromosomal localization and replication event of the LaWRKY family members

**Figure 4:**
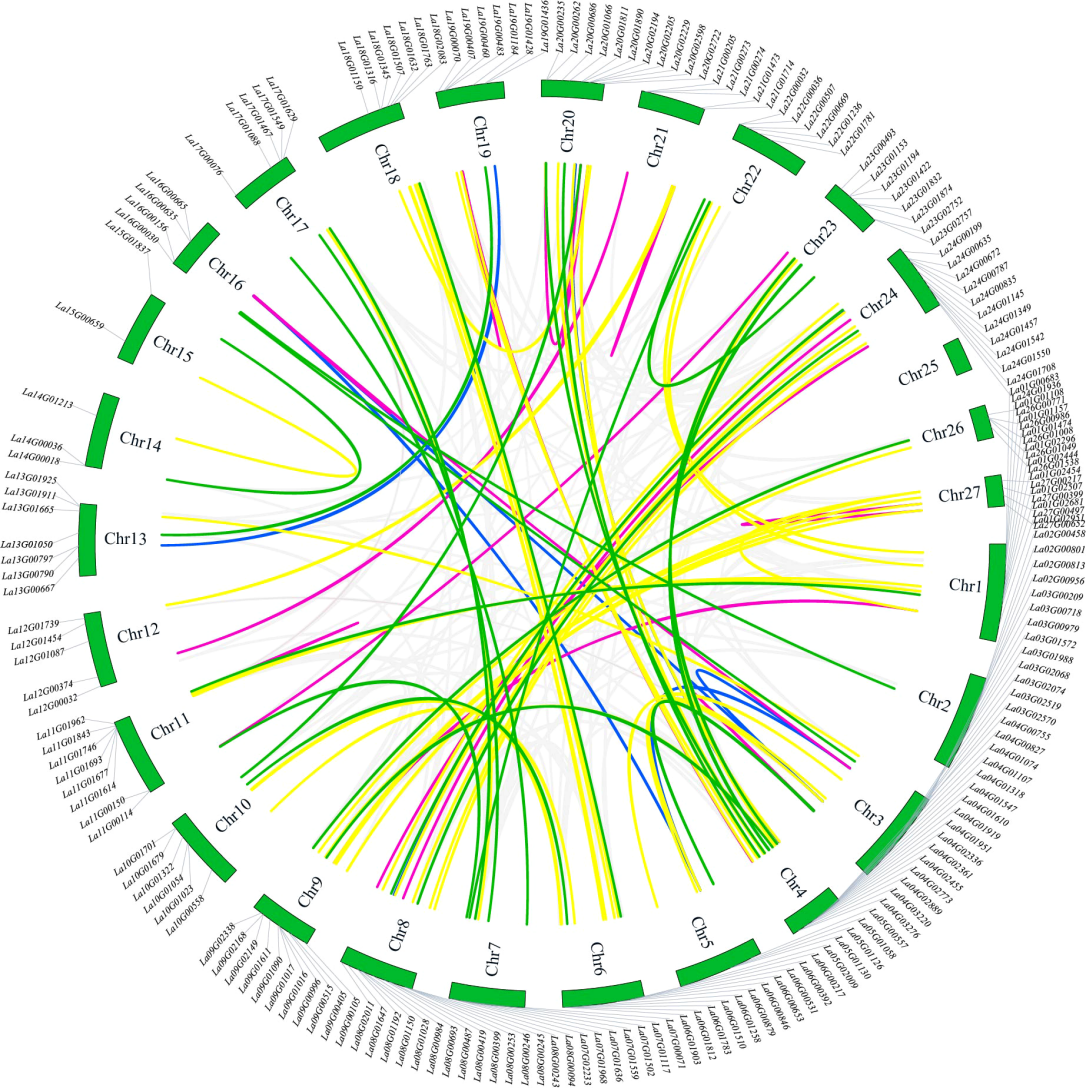
Chromosomal localization and repetitive events in *LaWRKY* genes of *L. angustifolia*. Gene pairs with varying degrees of homology are visually represented by colored lines in the diagram. The purple line signifies gene pairs with a homology of 96-100%, the yellow line represents gene pairs with a homology of 90-95%, the green line indicates gene pairs with homology ranging from 85% to 89%, and the blue line denotes gene pairs with 80-84% homology.

Based on the data depicted in **Figure 4**, the chromosomal mapping of the *LaWRKY* gene was conducted to examine its gene duplication pattern. Among the 207 total *LaWRKY* genes, 194 were successfully mapped to 26 chromosomes (Chr1~Chr27), except for Chr25. The uneven distribution of *LaWRKY* genes across chromosomes was observed, with Chr4 and Chr8 containing a relatively higher number of genes (16 and 15, respectively), while Chr15 only harbored two *LaWRKY* genes.

Within the *LaWRKY* dataset, a total of 92 gene pairs exhibited gene duplication events. Among these pairs, 58 demonstrated homology exceeding 90%. Significantly, eight pairs of genes, such as *La03G02519* and *La03G02570*, *La16G00635* and *La16G00665*, *La21G01473* and *La21G01714*, *La27G00497* and *La27G00652*, exhibited homology levels between 99% to 100%. These gene pairs were located on separate chromosomes, suggesting the occurrence of tandem gene duplication. Additionally, seven pairs of genes, including *La16G00665* and *La03G00979*, *La19G00460*, and *La04G03276*, shared homology of 96% to 98.99%. However, these gene pairs were distributed on different chromosomes, indicating a potential duplication event. The 25 remaining pairs of genes exhibited homology levels ranging from 85% to 89.99%, while 9 pairs fell within the range of 80 to 84.99%. Except for *La12G00374* and *La21G00273*, the other gene pairs within this set originated from the same group, suggesting the potential generation of tandem repeats during evolution.

### 3.5 *LaWRKY* expression profile in different tissues of *L. angustifolia*

**Figure 5:**
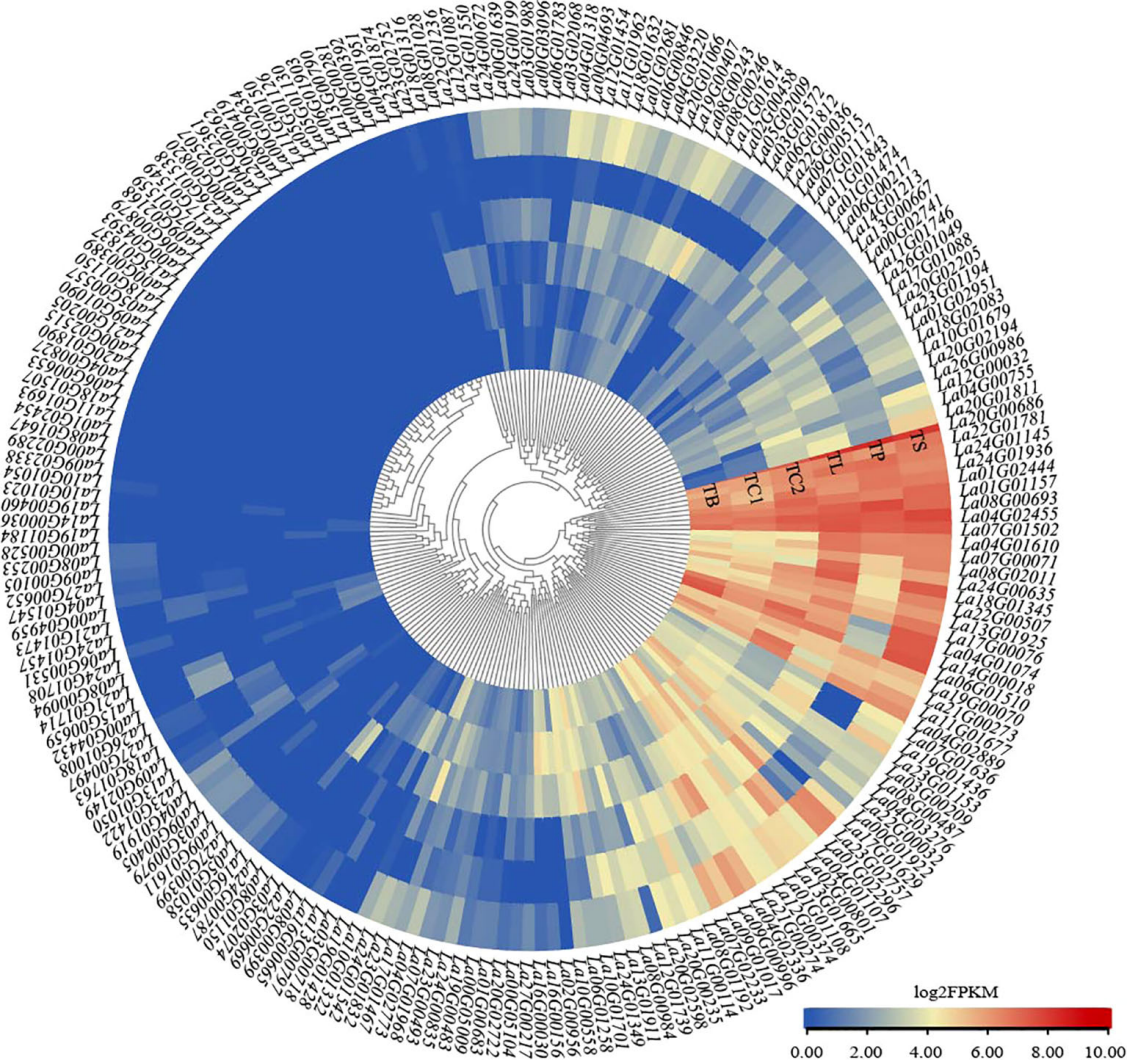
Heatmap of the expression profiles of *LaWRKY* genes in six different tissues, color bars indicate the expression value log2 (FPKM), and different colors indicate different expression levels. The different organizations in the Figure are TB, bud; TC1, fresh calyx; TC2, mature calyx; TL, leaf; TP, petal; TS, stem.

Transcriptome sequencing results indicated that all LaWRKY genes were expressed in at least one of the six tissues, with expression level (FPKM) greater than zero (**Figure 5**). Notably, 22 *LaWRKY* genes exhibited high expression across all six tissues (*La01G01157*, *La01G02444*, *La04G01074*, *La04G01610*, *La04G02455*, *La04G02889*, *La06G01510*, *La07G00071*, *La07G01502*, *La08G00693*, *La08G02011*, *La11G01677*, *La13G01925*, *La14G00018*, *La17G00076*, *La18G01345*, *La19G00070*, *La20G00686*, *La21G00273*, *La22G00507*, *La22G01781*, *La24G01145*, and *La24G01936*) (FPKM>2), suggesting their potential significance in the growth and development of *L. angustifolia*. *La04G01074*, *La06G01510*, *La14G00018*, and *La23G02757* exhibited high expression levels across all tissues, except for petals. Additionally, *La02G00956*, *La03G00718*, *La19G00483*, *La27G00217*, *La23G01832*, *La16G00156*, *La10G01322*, *La16G00030*, *La17G01629*, *La10G00558*, *La06G01258*, and *La04G03276* displayed elevated expression in flower buds and calyx, particularly in the young calyx where the expression level was notably prominent. Furthermore, a total of 24 genes, including *La04G02336*, *La09G00996*, *La02G00458*, *La19G01436*, *La10G01679*, and *La23G01153*, exhibited significant expression in the stems and leaves of *L. angustifolia*. Conversely, eight genes, including *La02G00801*, *La15G00659*, and *La00G04432*, demonstrated higher expression exclusively in the petals, further highlighting the specificity of these genes in the developmental process of specific tissues.

### 3.6 Prediction and analysis of *LaWRKY* gene promoter cis-acting elements

The analysis conducted in this study focused on 15 promoter elements derived from the *LaWRKY* gene family (**Supplementary Figure 3A**), including MBS (MYB binding site), LTR (low-temperature response element), TC-rich (defense and stress elements), ABRE (abscisic acid response), W-box (WRKY binding site), which were involved in the MeJA reaction (CGTCA-motif, TGACG-motif), P-box (gibberellin reaction), ARE (anaerobic induction), TCA-element (salicylic acid reaction), TGA (auxin response), and four photoreaction elements (TCT-motif, ATCT-motif, ACE, LAMP-element). It was observed that each member of the *LaWRKY* gene family was regulated by multiple factors. According to the findings presented in **Supplementary Figure 3B**, the MeJA reaction and abscisic acid were found to be the most significant components, followed by the light reaction, anaerobic induction, low-temperature response, and W-box. These results suggest that *LaWRKY* plays a crucial role in the response of *L. angustifolia* to abiotic stress, with a complex network of mutual regulation of expression and functional execution among each *LaWRKY* gene.

### 3.7 Expression analysis of *LaWRKY* gene after multiple hormone treatments and abiotic stresses

**Figure 6:**
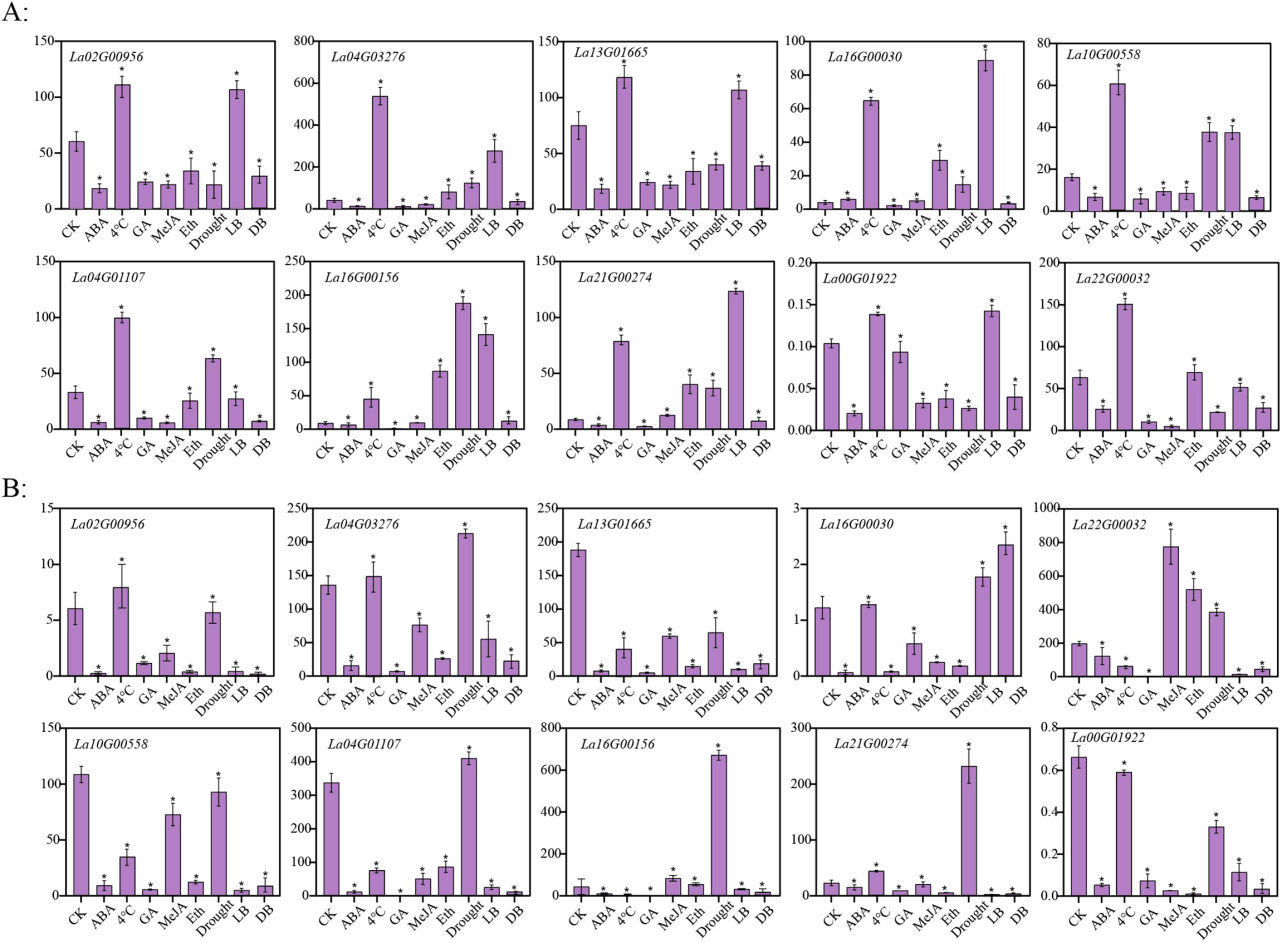
Expression patterns of 10 *LaWRKY* genes in *L. angustifolia* flower buds **(A)** and leaves **(B)** under different treatments. The vertical axis depicts the relative expression level, with the horizontal axis representing various treatments including CK (no processing), ABA (abscisic acid treatment), 4°C (cold treatment), GA (gibberellin treatment), MeJA (methyl jasmonate treatment), Eth (ethylene treatment), Drought (drought treatment), LB (intense light culture), and DB (dark culture). Statistical significance is denoted by asterisks (*), indicating a *p*-value of less than 0.001.

In order to investigate the potential role of *LaWRKY* in response to abiotic stresses and hormonal treatments, the expression levels of 10 *LaWRKY* genes in flower buds and leaves of *L. angustifolia* were examined following different treatments (**Figure 6**). Upon application of hormones to the flower buds, it was noted that, with the exception of *La16G00030*, which exhibited an upregulation in response to ABA treatment, the other genes displayed downregulation. Moreover, the expression levels of *La16G00030*, *La21G00274*, and *La16G00156* showed a slight increase after treatment with MeJA, while significant upregulation was observed in these three genes as well as *La22G00032* and *La04G03276* after treatment with ethylene. Additionally, it was observed that all 10 genes displayed upregulation following exposure to 4°C cold treatment, but downregulation after treatment with GA. In addition, the expression levels of *La16G00030*, *La21G00274*, *La16G00156*, *La04G03276*, *La10G00558*, and *La04G01107* were found to be induced by drought treatment. A comparative examination of the expression profiles of these 10 genes in flower buds under light and dark culture conditions revealed significant differences among all genes. Notably, the genes exhibited a marked increase in expression levels following exposure to intense light treatment, except for *La22G00032* and *La04G01107*, which displayed a slight decrease. Conversely, all genes displayed decreased expression levels after being treated in darkness.

The expression patterns of these 10 genes in leaves were observed to exhibit significant differences compared to the control, mirroring the patterns observed in flower buds after exposure to hormones and abiotic stresses. After treatments with ABA and GA, the expression of all genes was significantly reduced. *La22G00032* showed a significant increase in expression after MeJA and Eth treatments. *La02G00956*, *La04G03276*, *La16G00030*, and *La21G00274* showed an upward trend following cold leaf treatment. *La04G03276*, *La04G01107*, *La16G00156*, and *La21G00274* showed upregulated expression after drought treatment. Only *La16G00030* expression was upregulated during light and dark culture in leaves.

### 3.8 Functional analysis of *LaWRKY57* and *LaWRKY75* overexpressed in tobacco

**Figure 7:**
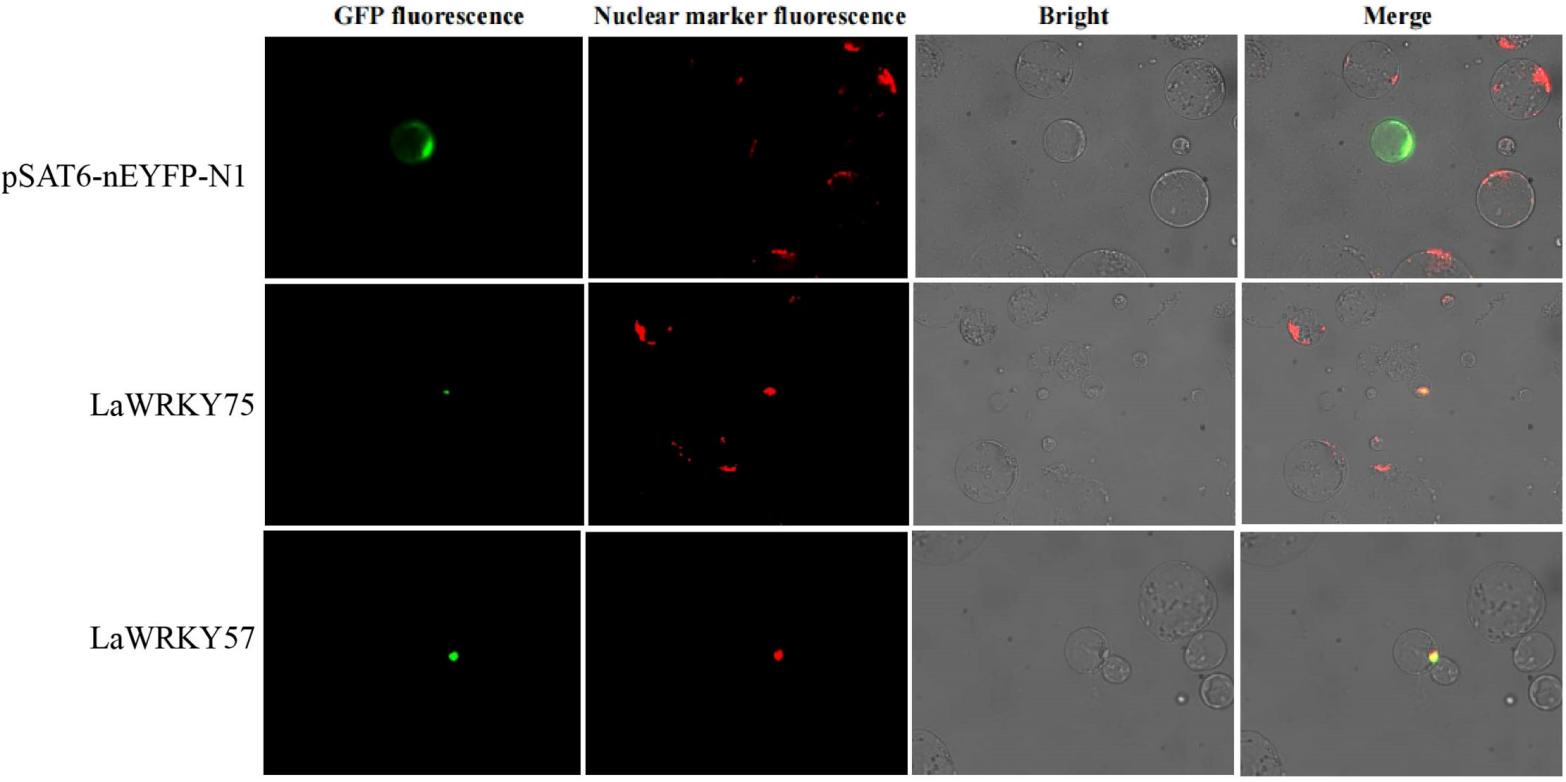
Subcellular localization analysis of LaWRKY57 and LaWRKY75. The EYFP fluorescence of LaWRKY57 and LaWRKY75 overlapped with the signal of the nuclear marker, confirming their nuclear localization.

**Figure 8:**
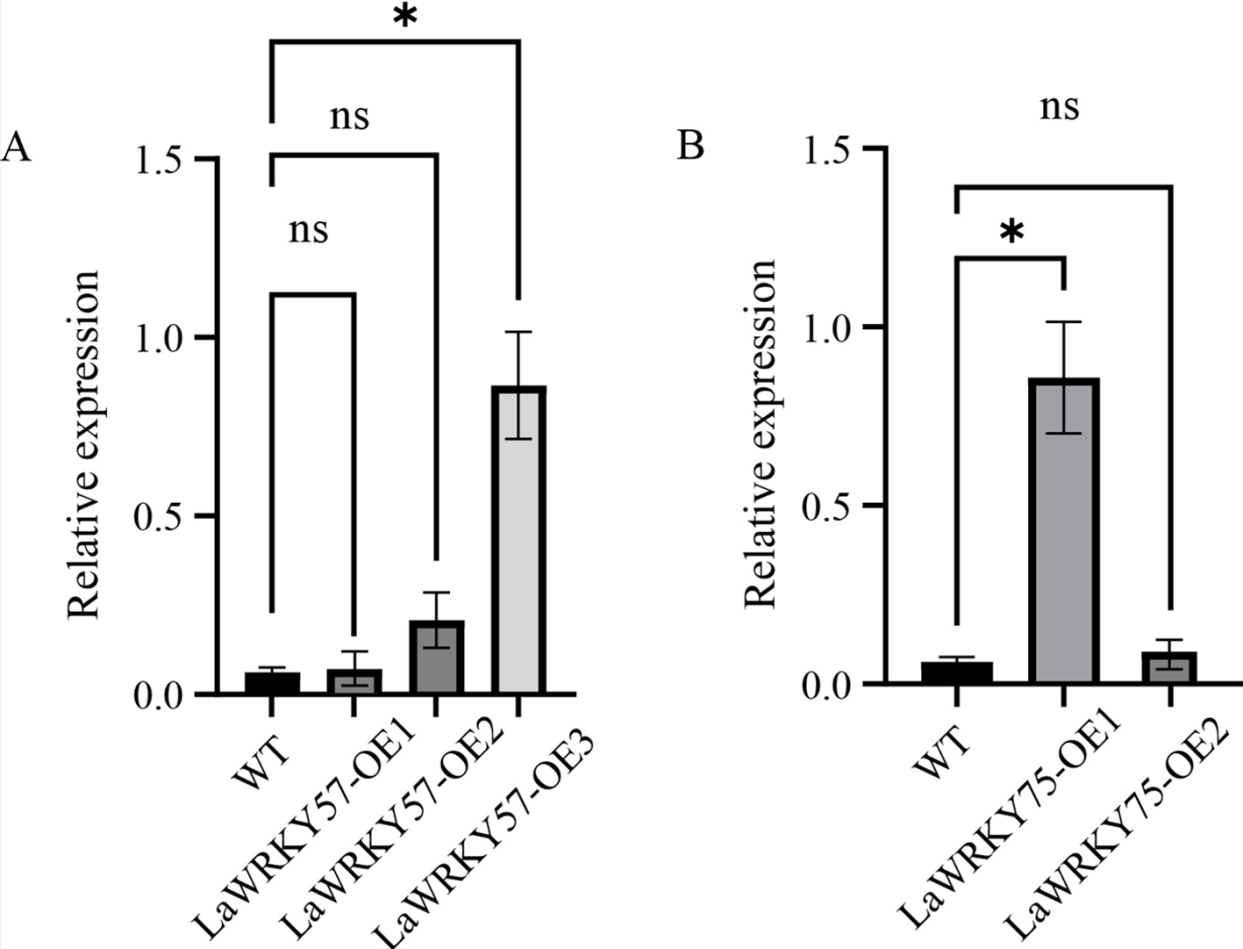
Expression analysis of *LaWRKY57* and *LaWRKY75* in WT and over-expressed transgenic lines of LaWRKY57 **(A)**, named *LaWRKY57*-OE1~OE3, and LaWRKY75 **(B)**, named *LaWRKY75*-OE1~OE2. Statistical significance is indicated by asterisks (*), representing a p-value of less than 0.001. "ns" denotes that there is no significant difference between groups.

**Figure 9:**
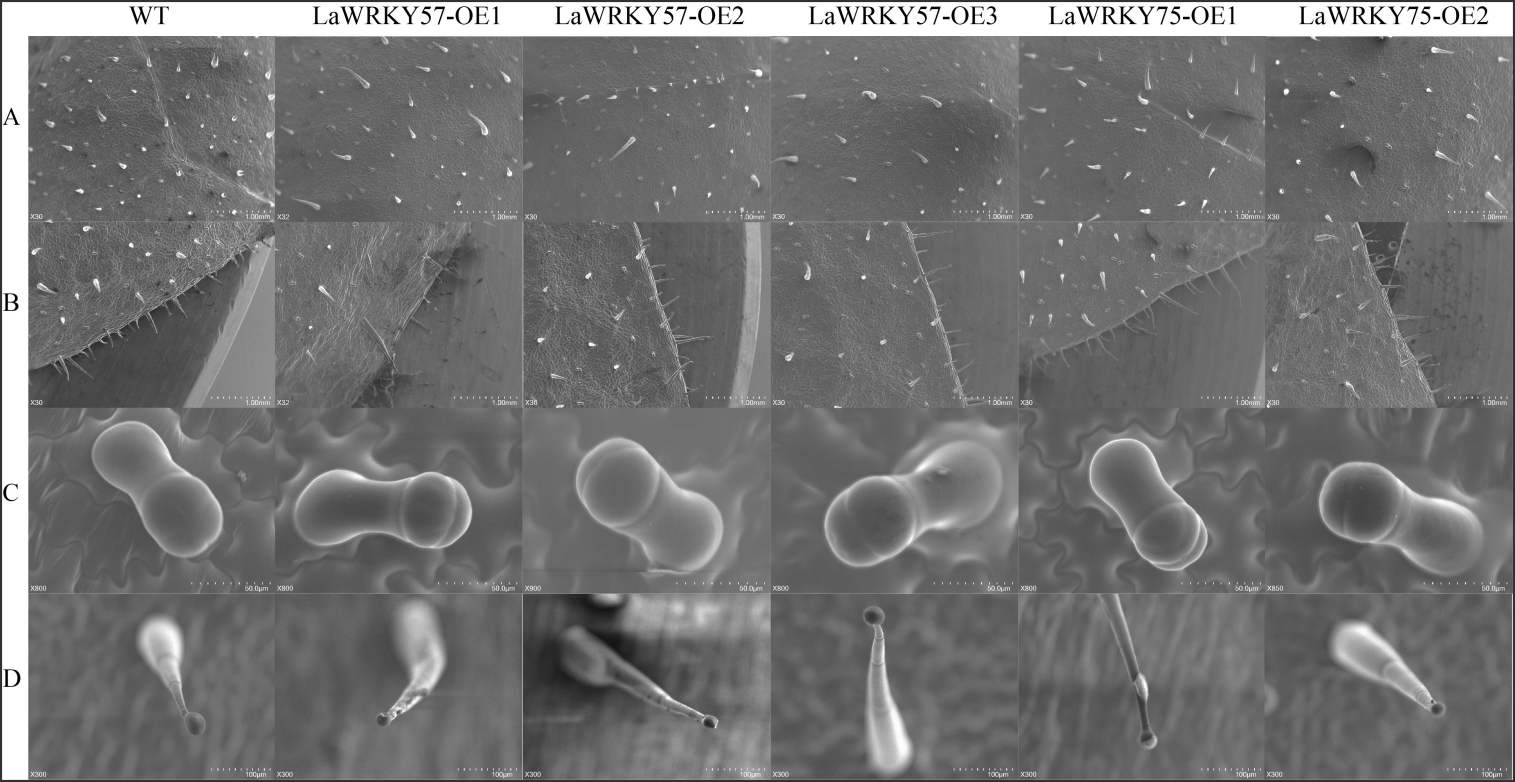
Scanning electron microscope (SEM) analysis of the surface of leaves in transgenic tobacco plants. **(A, B)** The glandular secretory trichome (GST) density was changed with the altered LaWRKY57 and LaWRKY75 expression in transgenic tobacco plants. **(C, D)** show short stalked glandular trichomes and long stalked glandular trichomes, respectively.

**Figure 10:**
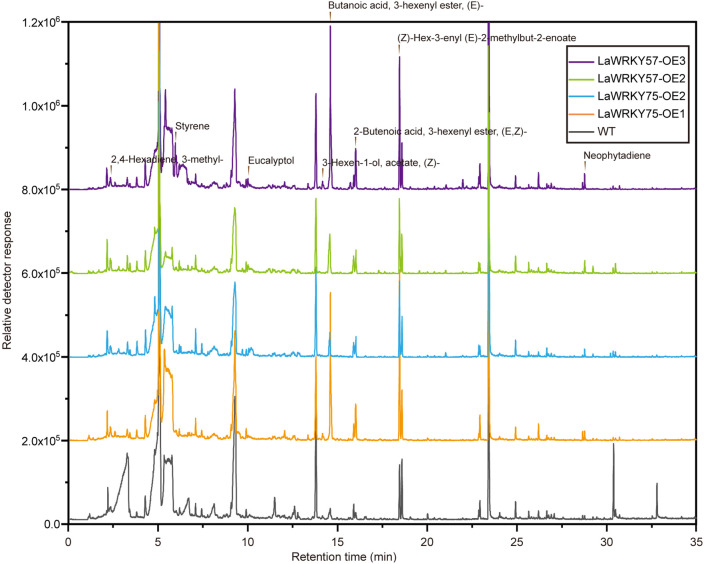
GC-MS analysis of the volatile terpenoids content in transgenic tobacco plants.

Subcellular localization analysis in lettuce protoplasts revealed nuclear fluorescence signals for both LaWRKY57 and LaWRKY75, suggesting their involvement in nuclear processes (**Figure 7**). After genetic transformation, three transgenic lines of *LaWRKY57* and two lines of *LaWRKY75* were identified. RT-qPCR analysis demonstrated that the expression levels of the target genes in these transgenic strains was higher than that in the wild-type (WT) plants (**Figure 8**). Specifically, the expression levels of *LaWRKY57* in LaWRKY57-OE3 and *LaWRKY75* in LaWRKY75-OE1 exhibited an approximate 20-fold increase relative to WT tobaccos. Furthermore, scanning electron microscopy showed a significantly reduced density of glandular trichomes on the leaves of these transgenic strains compared to WT tobacco (**Figures 9A, B**). The predominant types of glandular trichomes observed were short stalked glandular trichomes (**Figure 9C**) and long stalked glandular trichomes (**Figure 9D**), both of which were identified as secretory glandular trichomes. We chose the lines *LaWRKY57*-OE2 and OE3, *LaWRKY75*-OE1 and OE2 for the analysis of volatile terpenoids using GC-MS technology. The results revealed distinct differences between the transgenic lines and WT plants. Specifically, the transgenic lines over expressed *LaWRKY57* and *LaWRKY75* lacked 1-Hexanol, 5-methyl-2-(1-methylethyl)- a compound present in WT samples. Conversely, the transgenic lines produced unique compounds, including Eucalyptol and cis-3-Hexenyl iso-butyrate, which were absent in WT samples, as shown in **Figure 10**. The content of Neophytadiene significantly increased in these transgenic strains. Additionally, D-Limonene was detected in the LaWRKY57 line, whereas 3-Hexen-1-ol, acetate, (Z)- was produced in the LaWRKY75 line, suggesting a notable influence of *LaWRKY57* and *LaWRKY75* on the formation of glandular trichomes and the terpenoid profile in transgenic plants.

## 4 Discussion

The WRKY gene family, a unique class of transcription factors exclusive to plants, has been extensively studied and analyzed in various species (Liu et al., 2021). Herein, a total of 207 members belonging to the *L. angustifolia* WRKY family were successfully identified, surpassing the numbers observed in other plant species such as *Arabidopsis* and *P. trichocarpa* (Wang et al., 2011; Song and Gao, 2014). This discrepancy in gene family size may be attributed to *L. angustifolia*’s occurrence of two additional whole-genome duplications following the dicot γ triploid event. Furthermore, the comprehensive analysis of their whole genome revealed the presence of 55,696 pairs of homologous genes (Li et al., 2021).

### 4.1 The particularity of LaWRKY phylogeny and structure

The alignment of amino acid sequences revealed that within group I, La12G00374 and La21G00274 possessed solely the N-terminal WRKY domain, suggesting the possibility of either the loss of the C-terminal WRKY domain during evolution or an erroneous annotation in the genome sequence (Wang et al., 2019). Furthermore, the evolutionary classification demonstrated that the group IIc subgroup exhibits the highest number of members, which is consistent with findings in *Camellia sinensis* (Wang et al., 2019). In this study, four WRKY domain mutants were identified in groups I, IIc, and III, which potentially exhibited distinct binding specificity and biological functions due to modifications in DNA binding affinity (Cao et al., 2022). Importantly, variations in gene structure hold significant potential for elucidating the evolutionary history of the gene family, as highlighted by a previous study (Wang et al., 2019). In the present study, the number of *LaWRKY* introns ranged from 0 to 10, showing a wide spectrum of intron diversity. This range surpassed that observed in *Manihot esculenta* (1-5) (Wei et al., 2016) and *Sorghum bicolor* (0-7) (Baillo et al., 2020), indicating a high degree of variability in the gene structure of *LaWRKY*. Notably, *La06G00879* lacks introns, a phenomenon previously observed in genes reported in *Oryza sativa* (Wei et al., 2016) and *Arabidopsis* (Roy and David, 2007). This absence of introns may be attributed to the duplication of existing intron less genes, such as whole-genome duplication or tandem duplication, and the reverse transcription of genes containing introns (Zou et al., 2011).

### 4.2 *LaWRKY* chromosome distribution and genome replication events

The study conducted by Wu et al. (2021) delved into the evolution of gene families, a complex process influenced by mechanisms such as segmental duplication and tandem duplication concurring during whole genome replication. Within the *LaWRKY* gene family, 92 pairs of *LaWRKY* genes exhibited significant homology, suggesting that fragmental replication, rather than tandem duplication, primarily fueled the expansion of the *LaWRKY* gene family. Additionally, the presence of 26 tandem duplications in *LaWRKY* was noted, wherein two or more genes were located on the same chromosome. However, it was found that tandem repeat events contributed relatively little to the expansion of the *LaWRKY* gene family, a finding consistent with previous studies on *WRKY* genes in *Cucumis sativus* (Ling et al., 2011) and *Triticum aestivum* (Ning et al., 2017). These findings collectively suggest that tandem duplication, fragmental duplication, and whole genome duplication events have significantly contributed to the expansion and evolutionary trajectory of the *LaWRKY* gene family. Moreover, expression analysis revealed that a substantial proportion of *LaWRKY* genes exhibited low expression levels, implying that despite the family’s expansion, not all genes were functionally active. This observation aligns with the general principle that gene expression is intricately linked to gene function (Liu et al., 2021). Furthermore, like other gene families, the *LaWRKY* gene family exhibits tissue-specific variations in expression levels, as evidenced by studies conducted on *Solanum tuberosum* (Wu et al., 2021), *Cucumis sativus* (Chen et al., 2020), and *Maninot esculenta* (Wei et al., 2016) WRKY. Notably, two *LaWRKY* genes (L04G02336 and La09G00996) that exhibit high expression levels in leaves are phylogenetically related to *AtWRKY15* (AT2G23320) and *AtWRKY4* (AT1G13960), which are known to promote leaf growth in plants (Liu et al., 2021). This finding suggests that these *LaWRKY* genes may play a crucial role in the developmental processes of *L. angustifolia* leaves.

### 4.3 *LaWRKY* expression and response to different abiotic stresses

The expression pattern of *LaWRKY* gene in *L. angustifolia* transcriptome data exhibited tissue specificity, with 12 genes expressed in the calyx, and 24 genes in leaves and stems. Due to various types of glandular trichomes covering the leaves and calyx surfaces of *L. angustifolia*, they are the main tissues for synthesizing terpenoids (Zhang et al., 2023). Analysis of promoter sequences found cis-acting elements related to abiotic stress in the *LaWRKY* gene promoter, including ABRE, LTR, MeJA, TCA-element, P-box, ARE, and TGA-box, providing compelling evidence of the involvement of the *LaWRKY* gene in plant abiotic stress. Also, 10 *LaWRKYs* specifically expressed in the calyx and leaves was found that the expression of most selected *LaWRKY* genes in flower buds was induced by intense light and low temperature, while the expression in leaves was mainly induced by drought.

Over the years, numerous studies substantiated that intense light could stimulate the expression of transcription factors, consequently facilitating the production of diverse secondary metabolites. For instance, in *Arabidopsis*, the upregulation of *AtERF4* and *AtERF8* expression following light induction has been found to substantially augment the anthocyanin content (Koyama and Sato, 2018). In *Thymus vulgaris* seedlings, light induction was found to trigger the synthesis of monoterpenoids, particularly thymol, the primary constituent of essential oils, which exhibited an increase in production with prolonged irradiation time (Sharafzadeh, 2012). After undergoing light treatment, the apple transcription factor *MdWRKY1* exhibited a positive regulatory role in the long chain non-coding RNA (lncRNA) *MdLNC499* to further adjust the expression of *MdERF109*, ultimately leading to a significant increase in anthocyanin accumulation (Ma et al., 2021). The current investigation revealed that, with the exception of *La22G00032* and *La04G01107*, the expression levels of the remaining eight genes were elevated in response to light, while only *La16G00030* displayed upregulation in leaves of *L. angustifolia*. The heightened induction of several *LaWRKY* genes in the calyx of *L. angustifolia*, known to regulate terpenoid biosynthesis, was particularly prominent under intense light. These findings suggest a potential correlation between intense light and heightened essential oil production in flowers. Notably, the synthesis of terpenoids in flower buds appears to be particularly influenced by light stimulation. In regions characterized by intense sunlight, the presence of plant glandular trichomes can enhance light reflection, and improve resistance to UV radiation and low temperatures (Sun et al., 2020). This feature is indicative of the primary structure involved in essential oil synthesis, which may explain why the diameter of *L. angustifolia* calyx is greater than that of leaf glandular trichomes (Zhang et al., 2023). Consequently, it can be inferred that the Mediterranean region and the Yili, Xinjiang, China are the primary production areas for *L. angustifolia* (Dong et al., 2020), as the local high light intensity provides optimal conditions for essential oil synthesis in *L. angustifolia* plants.

WRKY are integral components of hormone signaling pathways (Jiang et al., 2017). In the abscisic acid (ABA) signaling pathway of *Arabidopsis*, *AtWRKY40* functions as a central negative regulator, modulating downstream gene expression through interactions with ABI4 and ABI5 (Shang et al., 2010). The integration of ABA, gibberellin (GA), and light signaling pathways further regulates seed germination by modulating the key regulatory hub, ABI5 (Zhao et al., 2022). In the present study, it was observed that ABA and GA significantly suppressed the expression of several *LaWRKY* genes, whereas Eth induced their expression. These results suggest that *LaWRKY* may play a role in plant growth and development, as well as in the response to external stressors such as intense light and cold, through ABA, GA, and Eth-dependent signaling pathways.

### 4.4 *LaWRKY57* and *LaWRKY75* act as potential negative regulatory factors in the synthesis of terpenoids

Initial research on the roles of *LaWRKY57* and *LaWRKY75* revealed their nuclear localization, aligning with predictions generated by the WoLF PSORT website (**Supplementary Table 3**). Additionally, *McWRKY57*, a homolog from *Mentha Canada*, was found to be a nuclear-localized transcription factor that enhanced drought tolerance in transgenic Arabidopsis (Bai et al., 2023). Further investigation through overexpression in tobacco demonstrated their impact on glandular hair development and volatile terpenoid composition in transgenic tobacco leaves, potentially exerting a suppressive effect on terpenoid synthesis. Glandular trichomes serve as the primary site for the biosynthesis of monoterpenes, sesquiterpenoids, and other compounds, with alterations in their characteristics potentially impacting the terpenoid composition (Zhang et al., 2023). Following the overexpression of *LaWRKY57* and *LaWRKY75* in tobacco, a notable absence of 1-Hexanol, 5-methyl-2-(1-methylethyl)- volatile terpene components in leaves was observed. Conversely, Neophytadiene levels were markedly increased in comparison to wild-type tobacco, accompanied by the emergence of novel compounds such as Eucalyptol and cis-3-Hexenyl iso-butyrate. Additionally, *LaWRKY57* and *LaWRKY75* were found to facilitate the synthesis of D-Limonene and 3-Hexen-1-ol, acetate, respectively. These findings suggest that *LaWRKY57* and *LaWRKY75* effectively modified the terpenoid profile of transgenic plants. Although previous research has not extensively examined this specific aspect, it has been found that in Arabidopsis, WRKY57 interacts with the JA signaling pathway inhibitor JAZ4/JAZ8 and the Auxin signaling pathway inhibitor IAA29, resulting in the negative regulation of jasmonic acid induced leaf senescence (Jiang et al., 2012, 2014). The observed decrease in glandular trichomes in genetically modified tobacco and the absence of key terpenoid components related to lavender, such as 1-Hexanol, 5-methyl-2-(1-methylethyl)-, suggest that LaWRKY57 may play a negative role in the regulation of terpenoid synthesis in lavender. This finding underscores the significant role of LaWRKY transcription factors in modulating the production and diversity of volatile terpenoids in lavender essential oil. Furthermore, it establishes a foundation for enhancing the quality of lavender essential oil and offers new insights into the synthesis and regulatory mechanisms of terpenoids in aromatic plants.

In summary, the *L. angustifolia* WRKY transcription factors have undergone various expansions including tandem repeats, segmental duplications, and whole genome duplications. As a result, the number of LaWRKY transcription factor family genes is 207, surpassing the count reported in multiple previously studied species. The expression patterns of these genes in different tissues of *L. angustifolia* are highly specific, indicating functional disparities among them. Notably, 12 gene members exhibit elevated expression levels in sepals, while 8 gene members demonstrate higher expression levels in petals. Additionally, 24 gene members exhibit high expression levels in both flowers and leaves. Promoter analysis revealed that various hormones and abiotic stresses influence the regulation of these genes. After conducting an analysis of the expression patterns of 10 genes in response to various treatments, it was noted that the stimulation of gene expression in flower buds was primarily influenced by intense light and low temperature, whereas gene expression in leaves was triggered by drought. Specifically, two genes, *LaWRKY57* and *LaWRKY75*, were identified as potential negative regulators in the biosynthesis of terpenoids. These results lay the foundation for further research on the biological function of LaWRKY in *L. angustifolia*.

## Data Availability

The original contributions presented in the study are publicly available. The RNA-seq data were deposited in GenBank with index number PRJNA892961.
