# Supplementary material for: Genome-wide investigation of WRKY gene family in Lavandula angustifolia and potential role of LaWRKY57 and LaWRKY75 in the regulation of terpenoid biosynthesis
Source: Front Plant Sci. 2024 Oct 8;15:1449299. doi: 10.3389/fpls.2024.1449299 (PMC11496791; doi:10.3389/fpls.2024.1449299)
Supplement: Supplementary file 1 [file DataSheet1.docx]

Genome-wide investigation of WRKY gene family in *Lavandula angustifolia* and potential role of *LaWRKY57* and *LaWRKY75* in the regulation of terpenoid biosynthesis

Kelaremu Kelimujiang^[[1]](#footnote-0)^, Wenying Zhang^2^, Xiaxia Zhang^1^, Aliya Waili^1^, Xinyue Tang^1^, Yongkun Chen^1*^, Lingna Chen^1*^

^1^Xinjiang Key Laboratory of Special Species Conservation and Regulatory Biology, Key Laboratory of Plant Stress Biology in Arid Land, School of Life Sciences, Xinjiang Normal University, Urumqi, 830054, China

^2^Key Laboratory of Plant Resources, Institute of Botany, Chinese Academy of Sciences, Beijing, 100093, China

*** Correspondence:**

Dr. Yongkun Chen, Xinjiang Normal University, Urumqi, 830054, China, [chenyk@xjnu.edu.cn](mailto:chenyk@xjnu.edu.cn)

Dr. Lingna Chen, Xinjiang Normal University, Urumqi, 830054, China, [chenln2003@1](mailto:ln.chen@xjnu.edu.cn)63.com

# Supplementary Figures and Tables

## Supplementary Figures


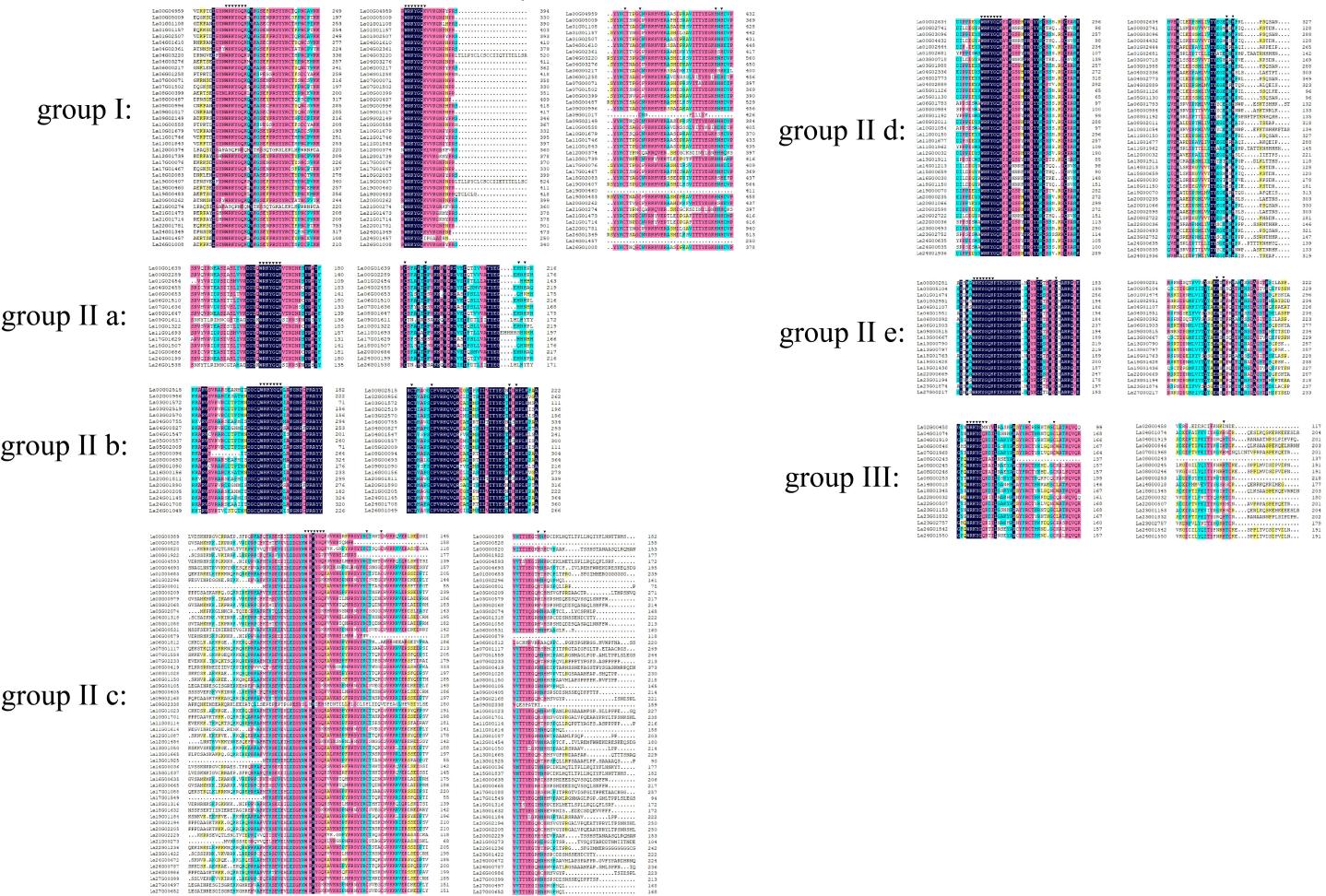


**Supplementary Figure 1.** Multiple sequence alignment of the WRKY domains in *L. angustifolia.* Th*e* multiple sequence alignment revealed the presence of highly conserved WRKYGQK and zinc finger motif sequences highlighted in red boxes.


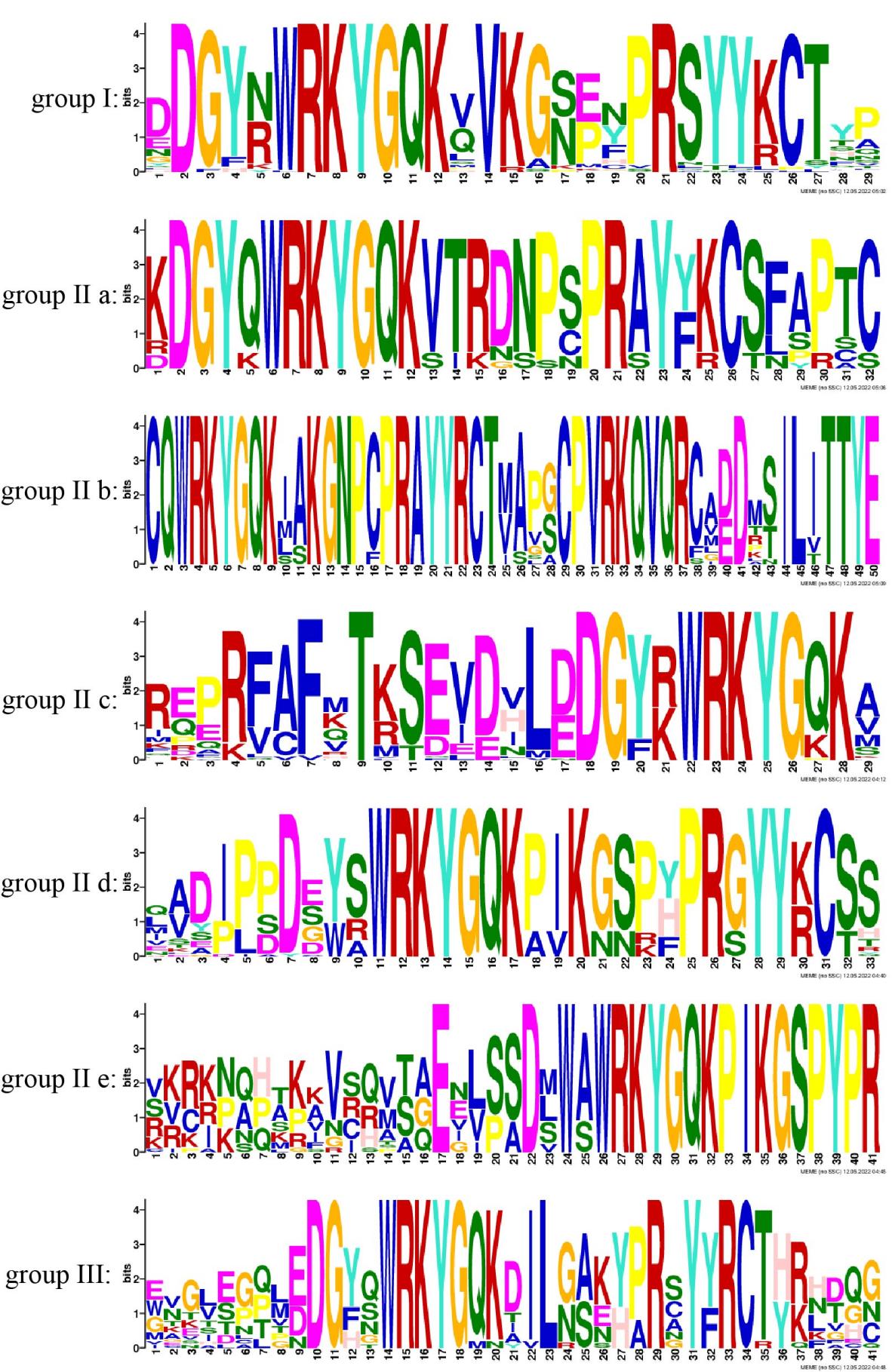


**Supplementary Figure 2**. The conserved domains within the LaWRKY protein sequence.

Note: The Y-axis, quantified in bits, illustrates the total height of the stacked symbols, denoting the level of sequence conservation at a particular position. Furthermore, the vertical position of the individual symbols within the stack reflects the proportional occurrence of each amino acid at that precise location.


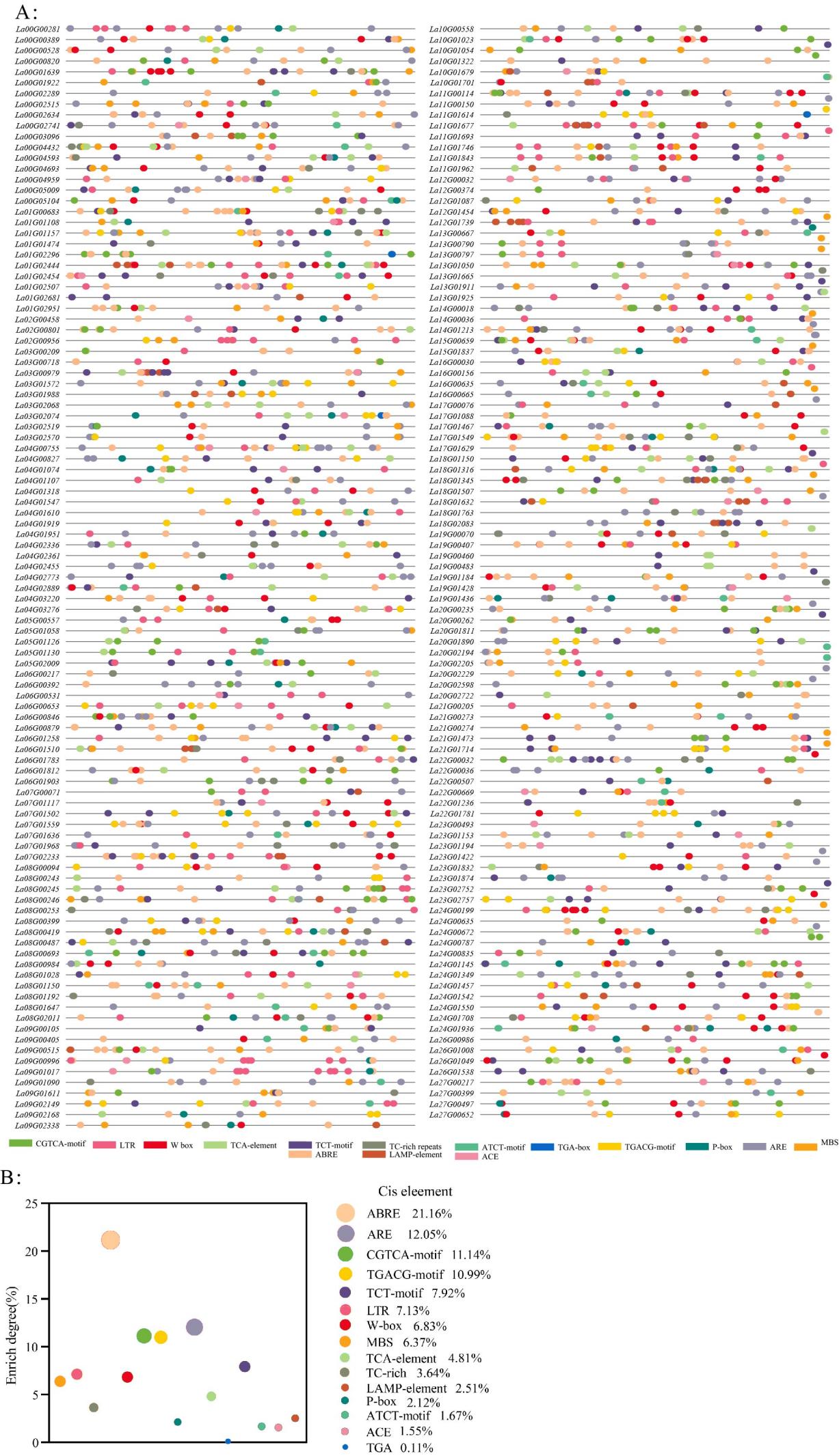


**Supplementary Figure 3.** A: *LaWRKY* gene promoter cis-element analysis, B: Proportion of each cis-element in the *LaWRKY* gene.

Note: The varying colors depicted in Figures A and B correspond to distinct cis-elements, while the diameter of the circles in Figure B is directly proportional to the relative abundance of promoter components.

## Supplementary Tables

Supplementary Table 1. Primers for RT-qPCR analysis.

| Gene ID | Primer (5’-3’) |
| --- | --- |
| *La02G00956* | F:ATCGAGAGGGTGGAGACGAA R:TTGGGCCATGATTTGAAGCG |
| *La04G03276* | F:ACCTGGGTGTTGGAATTGGG R:TGAAAGTTGCGAGCTTCCCT |
| *La10G00558* | F:GCAGTGATTGTGCACTTGGG R:GCAGTGTGTACTTCACCGGA |
| *La13G01665* | F:AGCTTCGCCTTCAGTTAGGG R:TGCTTTTTAGCCTTGCTCGC |
| *La16G00030* | F:AGTACGGCCAGAAAGCAGTC R:TTCTCCACCGGATGCGTATG |
| *La04G01107* | F:TAGCGGAGGTCGAGGATGAT R:TTCGAAAGCCAGGGGAACTG |
| *La21G00274* | F:CGACCTACCGTCAGCAACAT R:GGTTCTCTACCCGGCACAAT |
| *La00G01922* | F:TTAGATCTGCACCCACCACC R:GGTCCGTATTTCCGCCATTTG |
| *La16G00156* | F:CACCCAACATGGCCCACTTA R:TGCAACGGCAACCCTAAACT |
| *La22G00032* | F:CGGTCGTATCCGCTCAGAAT R:GGCGGGCCTCTTGTAACTAT |
| *LaActin* | F:GGTCGTACAACTGGTATTGTTC R:TCCAGCAGCTTCCATTCCGATCA |

Supplementary Table 2. Primers for gene cloning and vector construction.

| Gene ID | primer (5’-3’) |
| --- | --- |
| LC1665 | F:GGCAGCGGCCGAATTCATGGGCGGCAAGGAGAA R:CGGCCCCGGTGGATCCTCATTTCATCCGGGGCG CGGCCCCGGT |
| LC0030 | F:GGCAGCGGCCGAATTCATGGAGGATCTTGGTCCGAAG R:CGGCCCCGGTGGATCCTTAATGTACGTATACCTTAGTCA |
| EYFP1665 | F:GAGCTCAAGCTTCGAATTCATGGGCGGCAAGGAGAA R:GCCCGCGGTACCGTCGACGTCATTTCATCCGGGGCG CGGCCCCGGT |
| EYFP0030 | F:GAGCTCAAGCTTCGAATTCATGGAGGATCTTGGTCCGAAG R:GCCCGCGGTACCGTCGACGTTAATGTACGTATACCTTAGTCA |
| LCdete | F:CACTCTCGGCATGGACGAGCTGTA R:CGCCTATGATCGCATGATATTTGC |
| EYFPdete | F:GACGCACAATCCCACTATCC R:CTCAACACATGAGCGAAACCC |

Supplementary Table 3. Physicochemical properties and subcellular localization of LaWRKY protein.

| Gene ID | Group | Subcellular localization | Number of amino acids | Molecular weight (Da) | Theoretical pI |
| --- | --- | --- | --- | --- | --- |
| La00G04959 | Ⅰ | Nuclear | 475 | 51687.94 | 6.74 |
| La00G05009 | Ⅰ | Nuclear | 451 | 50130.81 | 6.9 |
| La01G01108 | Ⅰ | Nuclear | 474 | 51141.02 | 8.54 |
| La01G01157 | Ⅰ | Nuclear | 541 | 60123.09 | 6.69 |
| La01G02507 | Ⅰ | Nuclear | 474 | 51669.92 | 6.74 |
| La04G01610 | Ⅰ | Nuclear | 495 | 54146.25 | 6.38 |
| La04G02361 | Ⅰ | Nuclear | 593 | 64893.18 | 5.37 |
| La04G03220 | Ⅰ | Nuclear | 702 | 76609.61 | 5.81 |
| La04G03276 | Ⅰ | Nuclear | 554 | 60281.43 | 6.39 |
| La06G00217 | Ⅰ | Nuclear | 447 | 49131.49 | 7.67 |
| La06G01258 | Ⅰ | Nuclear | 530 | 58615.21 | 8.71 |
| La07G00071 | Ⅰ | Nuclear | 485 | 53287.5 | 8.08 |
| La07G01502 | Ⅰ | Nuclear | 391 | 43695.87 | 8.05 |
| La08G00399 | Ⅰ | Nuclear | 505 | 56396.44 | 8.87 |
| La08G00487 | Ⅰ | Nuclear | 656 | 70981.67 | 5.8 |
| La09G00996 | Ⅰ | Nuclear | 503 | 55035.47 | 8.29 |
| La09G01017 | Ⅰ | Nuclear | 448 | 48793.58 | 6.43 |
| La09G02149 | Ⅰ | Nuclear | 414 | 45779.07 | 7.68 |
| La10G00558 | Ⅰ | Nuclear | 479 | 52917.97 | 8.8 |
| La10G01679 | Ⅰ | Nuclear | 408 | 44712.51 | 6.76 |
| La11G01746 | Ⅰ | Nuclear | 517 | 56754.49 | 6.74 |
| La11G01843 | Ⅰ | Nuclear | 478 | 52342.72 | 8.16 |
| La12G00374 | Ⅰ | Nuclear | 614 | 67451.32 | 5.95 |
| La12G01739 | Ⅰ | Nuclear | 422 | 46237.73 | 9.34 |
| La17G00076 | Ⅰ | Nuclear | 488 | 54048.3 | 7.6 |
| La17G01467 | Ⅰ | Nuclear | 451 | 50130.81 | 6.9 |
| La18G02083 | Ⅰ | Nuclear | 484 | 53026.05 | 6.63 |
| La19G00407 | Ⅰ | Nuclear | 701 | 76455.32 | 5.55 |
| La19G00460 | Ⅰ | Nuclear | 411 | 44909.42 | 6.24 |
| La19G00483 | Ⅰ | Nuclear | 562 | 61151.51 | 7.03 |
| La20G00262 | Ⅰ | Nuclear | 615 | 67349.48 | 5.98 |
| La21G00274 | Ⅰ | Nuclear | 484 | 53181.64 | 5.45 |
| La21G01473 | Ⅰ | Nuclear | 431 | 47333.84 | 9.27 |
| La21G01714 | Ⅰ | Nuclear | 431 | 47333.84 | 9.27 |
| La22G01781 | Ⅰ | Nuclear | 1038 | 114431.11 | 8.34 |
| La24G01349 | Ⅰ | Nuclear | 647 | 70144.08 | 6.04 |
| La24G01457 | Ⅰ | Nuclear | 250 | 28061.06 | 6.45 |
| La26G01008 | Ⅰ | Nuclear | 408 | 45213.46 | 8.07 |
| La00G01639 | Ⅱa | Nuclear | 307 | 34260.31 | 9.26 |
| La00G02289 | Ⅱa | Nuclear | 267 | 29516.2 | 9.3 |
| La01G02454 | Ⅱa | Nuclear | 245 | 27590.91 | 8.88 |
| La04G02455 | Ⅱa | Nuclear | 315 | 34930.22 | 8.15 |
| La06G00653 | Ⅱa | Nuclear | 216 | 24637.64 | 8.47 |
| La06G01510 | Ⅱa | Nuclear | 320 | 35207.6 | 8.06 |
| La07G01636 | Ⅱa | Nuclear | 264 | 29560.22 | 6.46 |
| La08G01647 | Ⅱa | Nuclear | 266 | 29402.09 | 9.3 |
| La09G01611 | Ⅱa | Nuclear | 267 | 29802.21 | 5.27 |
| La10G01322 | Ⅱa | Nuclear | 323 | 35674.97 | 5.95 |
| La11G01693 | Ⅱa | Nuclear | 295 | 33213.39 | 5.79 |
| La17G01629 | Ⅱa | Nuclear | 256 | 28791.33 | 6.45 |
| La18G01507 | Ⅱa | Nuclear | 261 | 29594.23 | 8.62 |
| La20G00686 | Ⅱa | Nuclear | 313 | 34688.97 | 8.45 |
| La24G00199 | Ⅱa | Nuclear | 307 | 34260.31 | 9.26 |
| La26G01538 | Ⅱa | Nuclear | 270 | 30085.5 | 5.12 |
| La00G02515 | Ⅱb | Nuclear | 388 | 42030.44 | 9.56 |
| La02G00956 | Ⅱb | Nuclear | 426 | 47060.21 | 5.66 |
| La03G01572 | Ⅱb | Nuclear | 396 | 41794.27 | 9.25 |
| La03G02519 | Ⅱb | Nuclear | 347 | 38163.89 | 8.54 |
| La03G02570 | Ⅱb | Nuclear | 347 | 38163.89 | 8.54 |
| La04G00755 | Ⅱb | Nuclear | 503 | 54663.19 | 6.23 |
| La04G00827 | Ⅱb | Vacuolar | 472 | 50144.81 | 6.1 |
| La04G01547 | Ⅱb | Nuclear | 447 | 48854.37 | 6.06 |
| La05G00557 | Ⅱb | Nuclear | 519 | 56698.25 | 5.75 |
| La05G02009 | Ⅱb | Nuclear | 412 | 43571.09 | 9.32 |
| La08G00094 | Ⅱb | Nuclear | 430 | 46764.16 | 5.47 |
| La08G00693 | Ⅱb | Nuclear | 542 | 58837.61 | 8.13 |
| La09G01090 | Ⅱb | Nuclear | 422 | 45937.1 | 7 |
| La16G00156 | Ⅱb | Nuclear | 443 | 49104.54 | 6.09 |
| La20G01811 | Ⅱb | Nuclear | 510 | 55691.27 | 6.48 |
| La20G01890 | Ⅱb | Chloroplast | 502 | 53571.48 | 6 |
| La21G00205 | Ⅱb | Nuclear | 388 | 42118.59 | 9.54 |
| La24G01145 | Ⅱb | Nuclear | 542 | 58887.62 | 6.78 |
| La24G01708 | Ⅱb | Nuclear | 554 | 59645.51 | 5.53 |
| La26G01049 | Ⅱb | Nuclear | 447 | 48835.91 | 8.99 |
| La00G00389 | Ⅱc | Nuclear | 182 | 21039.51 | 6.65 |
| La00G00528 | Ⅱc | Nuclear | 158 | 17989.67 | 9.32 |
| La00G00820 | Ⅱc | Nuclear | 165 | 18475.28 | 6.08 |
| La00G01922 | Ⅱc | Nuclear | 177 | 19353.15 | 9.85 |
| La00G04593 | Ⅱc | Cytoplasmic | 172 | 19762.5 | 9.52 |
| La00G04693 | Ⅱc | Nuclear | 514 | 57741.28 | 8.97 |
| La01G00683 | Ⅱc | Nuclear | 338 | 38153.22 | 5.81 |
| La01G02296 | Ⅱc | Nuclear | 161 | 18356.11 | 5.41 |
| La02G00801 | Ⅱc | Chloroplast | 122 | 13632.21 | 9.14 |
| La03G00209 | Ⅱc | Chloroplast | 309 | 33947.56 | 7.01 |
| La03G00979 | Ⅱc | Nuclear | 217 | 24916.24 | 9.14 |
| La03G02068 | Ⅱc | Nuclear | 214 | 24617.93 | 9.07 |
| La03G02074 | Ⅱc | Nuclear | 168 | 19131.65 | 8.78 |
| La04G01318 | Ⅱc | Nuclear | 222 | 25176.66 | 6.79 |
| La05G01058 | Ⅱc | Nuclear | 213 | 24536.9 | 9.01 |
| La06G00531 | Ⅱc | Nuclear | 160 | 18480.63 | 6.53 |
| La06G00879 | Ⅱc | Nuclear | 118 | 13485.2 | 9.65 |
| La06G01812 | Ⅱc | Nuclear | 254 | 28001.1 | 9.48 |
| La07G01117 | Ⅱc | Nuclear | 333 | 37480.61 | 8.21 |
| La07G01559 | Ⅱc | Nuclear | 298 | 33957.56 | 6.93 |
| La07G02233 | Ⅱc | Nuclear | 260 | 29330.53 | 6.11 |
| La08G00419 | Ⅱc | Nuclear | 462 | 50740.74 | 7.14 |
| La08G01028 | Ⅱc | Nuclear | 273 | 30869.09 | 6.18 |
| La08G01150 | Ⅱc | Nuclear | 260 | 29718.72 | 6.84 |
| La09G00105 | Ⅱc | Nuclear | 165 | 19003.28 | 5.7 |
| La09G00405 | Ⅱc | Nuclear | 214 | 24734.56 | 8.25 |
| La09G02168 | Ⅱc | Nuclear | 264 | 29624.75 | 5.87 |
| La09G02338 | Ⅱc | Chloroplast | 159 | 18319.43 | 5 |
| La10G01023 | Ⅱc | Nuclear | 274 | 30486.63 | 7.17 |
| La10G01701 | Ⅱc | Nuclear | 301 | 32898.25 | 6.56 |
| La11G00114 | Ⅱc | Nuclear | 264 | 29838.03 | 5.41 |
| La11G01614 | Ⅱc | Nuclear | 158 | 17965.82 | 5.25 |
| La12G01087 | Ⅱc | Nuclear | 211 | 24260.1 | 8.96 |
| La12G01454 | Ⅱc | Nuclear | 499 | 55926.18 | 8.95 |
| La13G01050 | Ⅱc | Nuclear | 239 | 26745.71 | 8.63 |
| La13G01665 | Ⅱc | Nuclear | 265 | 29102.93 | 7.65 |
| La13G01925 | Ⅱc | Nuclear | 144 | 15727.61 | 8.91 |
| La14G00036 | Ⅱc | Nuclear | 177 | 20757.4 | 8.56 |
| La15G01837 | Ⅱc | Nuclear | 182 | 20996.48 | 6.37 |
| La16G00635 | Ⅱc | Nuclear | 206 | 23639.82 | 9.45 |
| La16G00665 | Ⅱc | Nuclear | 217 | 24975.27 | 9.13 |
| La17G01088 | Ⅱc | Nuclear | 337 | 38030.21 | 6.68 |
| La17G01549 | Ⅱc | Nuclear | 148 | 17123.28 | 8.85 |
| La18G01316 | Ⅱc | Cytoplasmic | 172 | 19773.48 | 9.52 |
| La18G01632 | Ⅱc | Nuclear | 172 | 20171.35 | 5.12 |
| La19G01184 | Ⅱc | Nuclear | 257 | 28824.85 | 7.18 |
| La20G02194 | Ⅱc | Nuclear | 313 | 33804.07 | 6.21 |
| La20G02205 | Ⅱc | Nuclear | 313 | 33804.07 | 6.21 |
| La20G02229 | Ⅱc | Nuclear | 165 | 18475.28 | 6.08 |
| La21G00273 | Ⅱc | Cytoplasmic | 126 | 14070.57 | 8.95 |
| La22G01236 | Ⅱc | Nuclear | 335 | 37801.83 | 5.41 |
| La23G01422 | Ⅱc | Nuclear | 222 | 25176.66 | 7.13 |
| La24G00672 | Ⅱc | Nuclear | 263 | 29612.51 | 8.97 |
| La24G00787 | Ⅱc | Nuclear | 296 | 32620 | 6 |
| La26G00986 | Ⅱc | Nuclear | 268 | 29928.11 | 5.56 |
| La27G00399 | Ⅱc | Chloroplast | 213 | 24669.4 | 7.7 |
| La27G00497 | Ⅱc | Nuclear | 168 | 19218.51 | 6.44 |
| La27G00652 | Ⅱc | Nuclear | 168 | 19200.47 | 6.44 |
| La00G02634 | Ⅱd | Nuclear | 328 | 36776.01 | 9.77 |
| La00G02741 | Ⅱd | Nuclear | 142 | 16325.31 | 9.54 |
| La00G03096 | Ⅱd | Nuclear | 289 | 32532.81 | 9.68 |
| La00G04432 | Ⅱd | Nuclear | 142 | 16502.51 | 9.37 |
| La01G02444 | Ⅱd | Nuclear | 269 | 29203.84 | 9.44 |
| La01G02681 | Ⅱd | Nuclear | 284 | 30993.29 | 6.26 |
| La03G00718 | Ⅱd | Nuclear | 190 | 21594.1 | 9.08 |
| La03G01988 | Ⅱd | Nuclear | 289 | 32452.74 | 9.58 |
| La04G02336 | Ⅱd | Nuclear | 310 | 33553.19 | 9.68 |
| La04G02773 | Ⅱd | Nuclear | 350 | 39227.44 | 9.81 |
| La04G02889 | Ⅱd | Nuclear | 337 | 36619.44 | 9.72 |
| La05G01126 | Ⅱd | Nuclear | 149 | 17522.19 | 10.71 |
| La05G01130 | Ⅱd | Mitochondrial | 97 | 11131.68 | 9.67 |
| La06G01783 | Ⅱd | Nuclear | 284 | 31648.92 | 4.92 |
| La08G00984 | Ⅱd | Nuclear | 269 | 30490.41 | 4.92 |
| La08G01192 | Ⅱd | Nuclear | 166 | 18126.85 | 8.56 |
| La08G02011 | Ⅱd | Nuclear | 325 | 35455.16 | 9.68 |
| La10G01054 | Ⅱd | Nuclear | 281 | 31299.81 | 5.3 |
| La11G00150 | Ⅱd | Nuclear | 328 | 36776.01 | 9.77 |
| La11G01677 | Ⅱd | Nuclear | 292 | 31723.64 | 9.64 |
| La11G01962 | Ⅱd | Nuclear | 259 | 28458.07 | 5.69 |
| La12G00032 | Ⅱd | Cytoplasmic | 228 | 25009.87 | 9.8 |
| La13G01911 | Ⅱd | Nuclear | 214 | 24437.2 | 8.13 |
| La14G01213 | Ⅱd | Nuclear | 142 | 16388.36 | 9.44 |
| La15G00659 | Ⅱd | Nuclear | 134 | 15536.43 | 9.63 |
| La16G00030 | Ⅱd | Nuclear | 205 | 23361.38 | 9.57 |
| La18G01150 | Ⅱd | Nuclear | 195 | 22386.15 | 8.93 |
| La19G00070 | Ⅱd | Nuclear | 327 | 35417.11 | 9.73 |
| La20G00235 | Ⅱd | Nuclear | 310 | 33447.92 | 9.62 |
| La20G01066 | Ⅱd | Nuclear | 331 | 37016.91 | 9.93 |
| La20G02598 | Ⅱd | Nuclear | 310 | 33421.84 | 9.62 |
| La20G02722 | Ⅱd | Nuclear | 195 | 22404.1 | 9.23 |
| La22G00036 | Ⅱd | Nuclear | 277 | 31138.9 | 5.97 |
| La23G00493 | Ⅱd | Nuclear | 322 | 36020.08 | 9.81 |
| La23G02752 | Ⅱd | Nuclear | 277 | 31107.73 | 6.28 |
| La24G00635 | Ⅱd | Nuclear | 252 | 27474.32 | 6.07 |
| La24G00835 | Ⅱd | Nuclear | 269 | 30516.45 | 4.95 |
| La24G01936 | Ⅱd | Nuclear | 326 | 35568.42 | 9.59 |
| La00G00281 | Ⅱe | Nuclear | 345 | 37252.73 | 4.78 |
| La00G05104 | Ⅱe | Nuclear | 333 | 37853.03 | 5.4 |
| La01G01474 | Ⅱe | Nuclear | 423 | 45307.11 | 5.96 |
| La01G02951 | Ⅱe | Nuclear | 315 | 34857.36 | 6.27 |
| La04G01107 | Ⅱe | Nuclear | 311 | 34509.17 | 6.41 |
| La04G01951 | Ⅱe | Nuclear | 319 | 35106.54 | 4.78 |
| La06G00392 | Ⅱe | Nuclear | 345 | 37294.81 | 4.78 |
| La06G01903 | Ⅱe | Nuclear | 403 | 43553.34 | 5.97 |
| La09G00515 | Ⅱe | Nuclear | 315 | 34859.33 | 6.27 |
| La13G00667 | Ⅱe | Nuclear | 314 | 35618.41 | 5 |
| La13G00790 | Ⅱe | Nuclear | 352 | 39140.71 | 7.7 |
| La13G00797 | Ⅱe | Nuclear | 348 | 38781.38 | 8.19 |
| La18G01763 | Ⅱe | Nuclear | 346 | 37308.75 | 4.7 |
| La19G01428 | Ⅱe | Nuclear | 335 | 37133.21 | 6.75 |
| La19G01436 | Ⅱe | Nuclear | 312 | 35381.16 | 5.3 |
| La22G00669 | Ⅱe | Nuclear | 414 | 43982.54 | 5.96 |
| La23G01194 | Ⅱe | Nuclear | 303 | 33768.48 | 6.6 |
| La23G01874 | Ⅱe | Nuclear | 334 | 36778.54 | 4.89 |
| La27G00217 | Ⅱe | Nuclear | 308 | 34080.64 | 6.83 |
| La02G00458 | Ⅲ | Nuclear | 194 | 22325.68 | 4.88 |
| La04G01074 | Ⅲ | Nuclear | 335 | 37347.21 | 5.22 |
| La04G01919 | Ⅲ | Nuclear | 316 | 35059.48 | 5.03 |
| La06G00846 | Ⅲ | Nuclear | 344 | 38356.7 | 5.52 |
| La07G01968 | Ⅲ | Nuclear | 340 | 38504.97 | 5.88 |
| La08G00243 | Ⅲ | Nuclear | 137 | 15213.31 | 9.43 |
| La08G00245 | Ⅲ | Nuclear | 285 | 32392.54 | 7.09 |
| La08G00246 | Ⅲ | Nuclear | 287 | 32597.83 | 6.66 |
| La08G00253 | Ⅲ | Nuclear | 308 | 33645.7 | 5.92 |
| La14G00018 | Ⅲ | Nuclear | 250 | 27823.39 | 6.44 |
| La18G01345 | Ⅲ | Nuclear | 344 | 38629.01 | 5.36 |
| La22G00032 | Ⅲ | Nuclear | 264 | 29468.86 | 6.7 |
| La22G00507 | Ⅲ | Nuclear | 245 | 28306.05 | 8.38 |
| La23G01153 | Ⅲ | Nuclear | 335 | 37423.73 | 5.49 |
| La23G01832 | Ⅲ | Nuclear | 322 | 35719.3 | 5.1 |
| La23G02757 | Ⅲ | Nuclear | 263 | 29578.93 | 6.5 |
| La24G01542 | Ⅲ | Nuclear | 285 | 32333.34 | 5.94 |
| La24G01550 | Ⅲ | Nuclear | 285 | 32333.34 | 5.94 |

1. [↑](#footnote-ref-0)
